# Genome Wide Characterization of Short Tandem Repeat Markers in Sweet Orange (*Citrus sinensis*)

**DOI:** 10.1371/journal.pone.0104182

**Published:** 2014-08-22

**Authors:** Manosh Kumar Biswas, Qiang Xu, Christoph Mayer, Xiuxin Deng

**Affiliations:** 1 Key Laboratory of Horticultural Plant Biology of Ministry of Education (MOE), Huazhong Agricultural University, Wuhan, Hubei, P.R. China; 2 Forschungsmuseum Alexander Koenig, Bonn, Germany; United States Department of Agriculture, United States of America

## Abstract

Sweet orange (*Citrus sinensis*) is one of the major cultivated and most-consumed citrus species. With the goal of enhancing the genomic resources in citrus, we surveyed, developed and characterized microsatellite markers in the ≈347 Mb sequence assembly of the sweet orange genome. A total of 50,846 SSRs were identified with a frequency of 146.4 SSRs/Mbp. Dinucleotide repeats are the most frequent repeat class and the highest density of SSRs was found in chromosome 4. SSRs are non-randomly distributed in the genome and most of the SSRs (62.02%) are located in the intergenic regions. We found that AT-rich SSRs are more frequent than GC-rich SSRs. A total number of 21,248 SSR primers were successfully developed, which represents 89 SSR markers per Mb of the genome. A subset of 950 developed SSR primer pairs were synthesized and tested by wet lab experiments on a set of 16 citrus accessions. In total we identified 534 (56.21%) polymorphic SSR markers that will be useful in citrus improvement. The number of amplified alleles ranges from 2 to 12 with an average of 4 alleles per marker and an average PIC value of 0.75. The newly developed sweet orange primer sequences, their *in silico* PCR products, exact position in the genome assembly and putative function are made publicly available. We present the largest number of SSR markers ever developed for a citrus species. Almost two thirds of the markers are transferable to 16 citrus relatives and may be used for constructing a high density linkage map. In addition, they are valuable for marker-assisted selection studies, population structure analyses and comparative genomic studies of *C. sinensis* with other citrus related species. Altogether, these markers provide a significant contribution to the citrus research community.

## Introduction

Tandem repeats (TR) are abundant elements in plant genomes. Evidence suggests that TR originate mainly from replication slippage events. Typically, slippage in a TR occurs about once every 1,000 generations, where slippage rates vary with repeat type, unit size as well as among species [1], [2]. TRs are usually classified according to their unit size into microsatellites, minisatellites, and satellites. There is no consensus regarding the unit size boundary between micro- and minisatellites. Proposed unit size ranges of microsatellites are e.g, 1–6 bp, 2–6 bp, 2–8 bp [3] and 1–10 bp. Microsatellites are also often referred to as simple sequence repeats (SSRs). In this study we consider repeat units of one to ten nucleotides as microsatellites. Their genomic abundance, co-dominant nature, easy assay, non-random distribution, correlation with many phenotypes as well as multi allelic feature have made microsatellites the marker of choice for diverse application in plant genetics. The availability of whole genomic sequences provides the opportunity to investigate the genome wide distribution, density, evolution and putative function of microsatellites. It is well known that the microsatellite frequency differs greatly among species [4] as well as among different genomic regions, *i.e.* introns, exons, CDS, intergeneric regions [5]. Previous studies demonstrated that the SSR distribution in genomic regions has practical implications with regard to their utility as molecular markers. Genic-SSR markers are more transferable to related species than genomic-SSRs. This feature helps to design anchor markers for comparative mapping studies. Since they are often more conserved, genic-SSRs may provide an insufficient degree of polymorphism to discriminate between closely related germplasm. Therefore genomic-SSRs may be valuable complements.

Sweet orange (*Citrus sinensis* [L.] Osbeck) is one of the most widely grown fruit crop in the world with a global production of 69.41 million tons and a total acreage of 4.06 million hectares in 2010. It contributes about 60% of the total world citrus production and consumption [6]. The remaining 40% are distributed among other citrus such as lemon, grape fruit and pummelo. Its value also stems from it being rich in vitamin C and other health beneficial elements. Most of the sweet oranges are diploids with a comparatively small genome size of about 367 Mb [7] having 9 chromosomes. Citrus taxonomy is fairly complex due to the unusual reproduction nature, nucellar embryony, a high frequency of bud mutations and wide cross-compatibility among the species [8], [9]. The high frequency of bud mutation is one of the causes for the narrow genetic basis of the cultivated citrus species. Inter species genetic diversity in sweet orange is relatively low compared to other citrus species as reveled in early studies using different types of molecular markers including RAPD, AFLP, ISSR (Inter simple sequence repeat), IRAP (Inter-retroelement amplified polymorphism) and SSRs [10]. The development of high density reference linkage maps of citrus is essential for the understanding of genome organization, evolution, tagging important quantitative trait loci (QTL) and map-based cloning of agronomical important traits. Consequently, it is necessary to develop a marker system in citrus that is highly polymorphic and user friendly. Among different marker systems, microsatellite markers are extensively used for genetic mapping and MAS studies in plant breeding. Unfortunately, the number of developed microsatellite markers is still low in citrus. Recently, Biswas et al. [9] and Ollitrault et al. [11] demonstrated the utility of BAC-end derived citrus SSR markers in linkage mapping and phylogenetic studies in citrus; while Chen el al. [12] established the utility of 100 citrus EST-SSR markers for genomic mapping analyses. Despite this progress in the total number of informative, robust and publicly available markers for citrus, their number is still insufficient for many important applications such as the construction of a high density linkage map, closely related cultivar identification, positional cloning, MAS, trait tagging and comparative mapping. The large scale development of these markers was not possible until the whole genomic sequence of citrus was available. In this study we surveyed, developed, and characterized SSR markers from the recently sequenced nuclear genomic sequence of sweet orange *cv* ‘Valencia’. Furthermore, we investigated the transferability of these markers to related species.

## Materials and Methods

### Plant materials

Sixteen genotypes were used for the wet lab verification and transferability analysis of the genome wide SSR primers. Plant materials were collected from the National Center of Citrus Breeding, Huazhong Agricultural University, Wuhan, China. In addition, these genotypes represent the major groups of citrus and its close relatives (Table S1 in File S1). Total genomic DNA was extracted from 5 g mature fresh young leaves of each genotype using the CTAB method [13] with subsequent RNase A treatment.

### Source of genomic sequences

The genome sequencing of a doubled-haploid callus line of sweet orange cv. ‘Valencia’ was performed with a whole-genome shotgun approach combined with the DNA-PET technology. The genome was assembled using the Short Oligo-nucleotide Analysis Package (SOAPdenovo). The high quality sequence reads were assembled into 4,811 scaffolds with N50 = 1.7 Mb. The total contig length (320.5 Mb) covers about 87.3% of the sweet orange genome. The frozen version of the sweet orange genome assembly, which consists of scaffolds and BAC end sequences, was used in this study for a genome wide SSR marker characterization.

### SSR mining and Primer design

The software SSRLocatorI V1.1 was used for genome wide SSR mining and primer design. To identify SSRs in the citrus genome, we searched for perfect repeats with a unit size of 1 to 10 bp and a length of at least 16 nucleotides. All SSRs were grouped into Class I (≥20 bp total length) and Class II (16–19 bp total length). Primer pairs were design to meet the following restrictions: the amplicon had to be in the range 150–500 bp, the primer annealing temperature was restricted to 55–60°C, the GC content had to be 40–60% and the primer length had to be 19–21 bp.

### *In silico* analysis of SSR polymorphism

*In silico* polymorphism analysis of SSR markers was perform using the virtual PCR strategy where pairs of primer sequences from Sweet Orange were mapped onto the Clementina genomic sequence scaffolds. (Only di- to hexanucleotide repeat primers where considered.) The specific *in silico*-generated amplicons from Clementina were compared with the expected amplicon size from Sweet Orange and their size differences were recorded. If an amplicon size differed by at least 2 bp, the SSRs was classified as polymorphic, while amplicons of identical size were considered as monomorphic. SSR loci with a 1-bp difference were considered ambiguous and were removed from the analysis.

### Functional annotation of genome wide SSR markers

An in house developed perl script was used to isolate flanking sequences of the SSR markers for assigning a putative function to each SSR marker. For primers in protein coding regions, this approach made use of the Blast2GO tool. The mapping and annotation of the sequences is based on sequence similarity according to gene ontology [14]. Therefore, sequences without BLAST hit have not been annotated. The default settings were used for the annotation parameters (E value filter of 1E-10 and annotation cutoff of 55).

### Wet lab verification and utility of the SSR markers

A total of 950 SSR markers with an average distance of 0.5 Mb were selected from the nine chromosomes for the wet lab validation, where a set of 16 citrus genotypes were used as targets species. PCR reactions were conducted as follows: 10 µl of PCR-volume consisted of 25 ng of genomic DNA, 1.5 mmol l^−1^ MgCl_2_, 0.2 mmol l^−1^ dNTPs, 1.0 U Taq DNA polymerase, 1× PCR buffer and 0.1 µmol l^−1^ of each primer pair. PCR amplification was conducted in a MJ-PTC-200 tm thermal controller (MJ Research, Waltham Mass) using the following program: 94°C for 5 min, 32 cycles at 94°C for 1 min, 55°C for 30 s, 72°C for 1 min, followed by a final step at 72°C for 4 min. After PCR, 8 µl of loading buffer (98% formamide, 2% dextran blue, 0.2 mM EDTA) was added to each sample. Samples were denatured at 90°C for 5 min and then immediately placed on ice. An aliquot (4 µl) of each sample was loaded onto 6% polyacrylamide gel (60 cm×30 cm×0.4 cm), which was run for 2 h and 30 min at 80 V. DNA bands were visualized with silver staining as described by Ruiz et al. [15]. The band size is reported for the most intense amplified band for each SSR or the average of the stutter if the intensity was the same. A 10 bp DNA ladder (Fermentas) was used as the reference point. Polymorphic information content (PIC) of each SSR was calculated using the following formula: 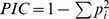, where p_i_ is the proportion of the *i*th allele.

### Gnome wide localization and database of SSR marker

A graphical presentation of the SSR marker distribution in different chromosomes of Sweet Orange was made using the MapChart 2.2 software. For an easy access and utilization of SSR markers, all markers were stored in a database, which will be made available to the public soon. Markers are named according to the following scheme: E.g. in M2H4Si3025, the M stands for the initial character of the developer name, the 2 depicts the chromosome and H stands for the host institute. 4 depicts the SSR unit size, Si the Citrus species (here *Citrus sinensis*) followed by the SSR pattern id, a number that is unique for each unit size.

## Results

### Frequency and distribution of SSR in the sweet orange genome

**Figure 1:**
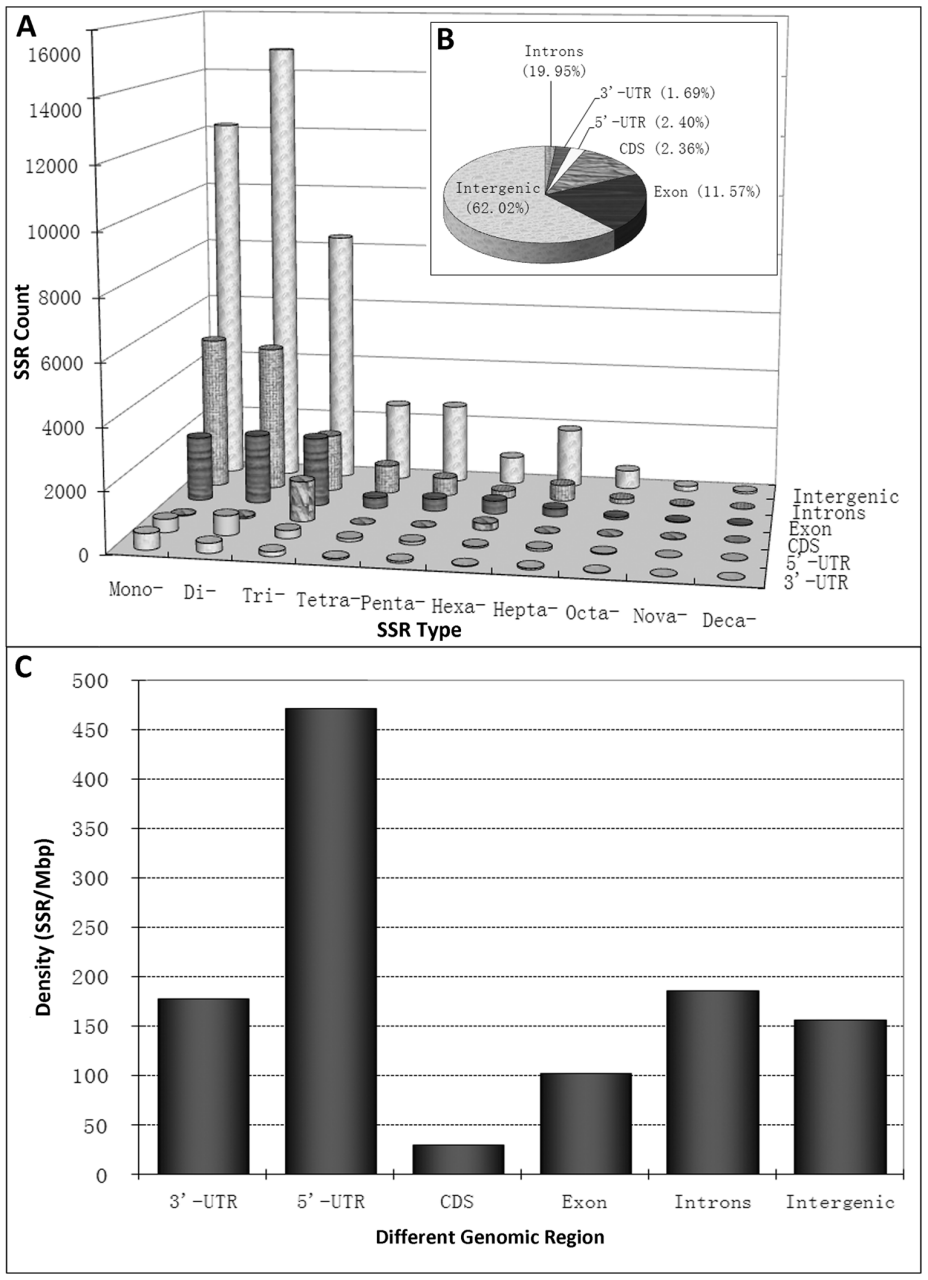
Absolute number count (A), relative number counts (B), and density (C) of SSRs in different genomic regions of the sweet orange genome.

In the first step, we analyzed the distribution and frequency of perfect microsatellites with a minimum length of 16-bp and a unit size of 1 to 10-bp. The results are summarized in Table 1. A total of 50,846 SSRs were identified which represents an overall density across the genome of 146.42 SSR/Mbp (i.e., one SSR found in every 6.8 kbp). Based on their length, SSRs are categorized into two classes, namely Class I (≥20 bp) and Class II (16–19 bp). Of the total number of SSR identified in the sweet orange genome, 28,211 (55.48%) were identified as Class I and 22635 (44.52%) as Class II. On average, the estimated frequency of Class I and Class II SSR was 81.24 and 65.18 per Mb, respectively. Altogether, the total lengths of mono- to deca-nucleotide repeats accounted for about 0.461% of the genome. Among the different unit sizes, dinucleotide repeats are the most common. They constitute 32.87% of all SSRs, followed by mono- (27.20%) and trinucleotide repeats (19.99%), while nona- and deca-nucleotide repeats were least abundant. Surprisingly, we found that hepta-nucleotide repeat numbers were two fold higher than hexanucleotides repeat numbers. Furthermore, we estimated the frequency distribution of SSRs in the nine chromosomes of the sweet orange genome and the result reveals that chromosome 4 has the highest frequency of SSRs (237.06 SSRs/Mb), while chromosome 8 contains the lowest (150.46 SSRs/Mb). Overall the frequency of SSRs in the different chromosomes was not significantly different (Table 2). SSR characteristics for different unit sizes are shown in Figure S1 and Table S2 in File S1. The mean number of trinucleotide (16.12) repeats is higher than others, except mononucleotides. The distribution of SSRs in different genomic region of the sweet orange genome is presented in Figure 1 and as a result it appears that SSR are denser in 5′-UTR region than in other genic regions.

**Table 1:** SSR characteristics in the genome assembly of *C. sinensis*.

SSR mining	Total	%
Total length of analyzed sequences (bp)	347267366	
Number of identified SSRs	50846	
Number of SSR Loci	46872	
SSR Frequency (Mbp)	146.42	
SSR Density (bp/Mbp)	4605.56	0.461
Distribution of SSRs		

Type	Class I*	Class II$	Total	%
Mononucleotides	3298	10530	13828	27.20
Dinucleotides	8882	7831	16713	32.87
Trinucleotides	5888	4274	10162	19.99
Tetra-nucleotides	2826	0	2826	5.56
Penta- nucleotides	2960	0	2960	5.82
Hexa- nucleotides	1194	0	1194	2.35
Hepta- nucleotides	2183	0	2183	4.29
Octa- nucleotides	704	0	704	1.38
Nona- nucleotides	193	0	193	0.38
Deca- nucleotides	83	0	83	0.16
Total (%)	28211(55.48)	22635(44.52)		
Frequency (SSR/Mb)	81.24	65.18		

* Class I :SSR loci are greater than 19 nt long;

$ ClassII: SSR loci smaller than 20 nt long.

**Table 2:** Chromosome wide distribution of SSRs in the *C. sinensis* genome.

SSR repeat class	Chromosome										Total
	1	2	3	4	5	6	7	8	9	Un	
Mononucleotides	1266	1395	1746	1333	1843	1031	1345	957	835	2077	13828
Dinucleotides	1578	1685	1914	1666	2015	1071	1834	1032	920	2998	16713
Trinucleotides	958	1005	916	858	1234	664	1075	706	588	2158	10162
Tetranucleotides	273	290	266	251	337	169	303	214	184	539	2826
Pentanucleotides	229	298	319	260	354	207	290	212	185	606	2960
Hexanucleotides	104	140	118	98	143	86	108	96	50	251	1194
Heptanucleotides	198	236	217	186	249	134	215	137	99	512	2183
Octanucleotides	54	70	76	54	81	37	60	48	40	184	704
Nonanucleotides	19	19	15	16	17	9	17	13	10	58	193
Decanucleotides	6	5	9	8	11	3	8	2	6	25	83
Size (Mb)	28.80	30.84	28.71	19.95	36.15	21.18	32.21	22.71	18.45	108.27	347.27
SSR/Mb	162.67	166.78	194.89	237.06	173.85	161.05	163.17	150.46	158.10	86.89	146.42
Total SSR	4685	5143	5596	4730	6284	3411	5255	3417	2917	9408	50846

**Figure 2:**
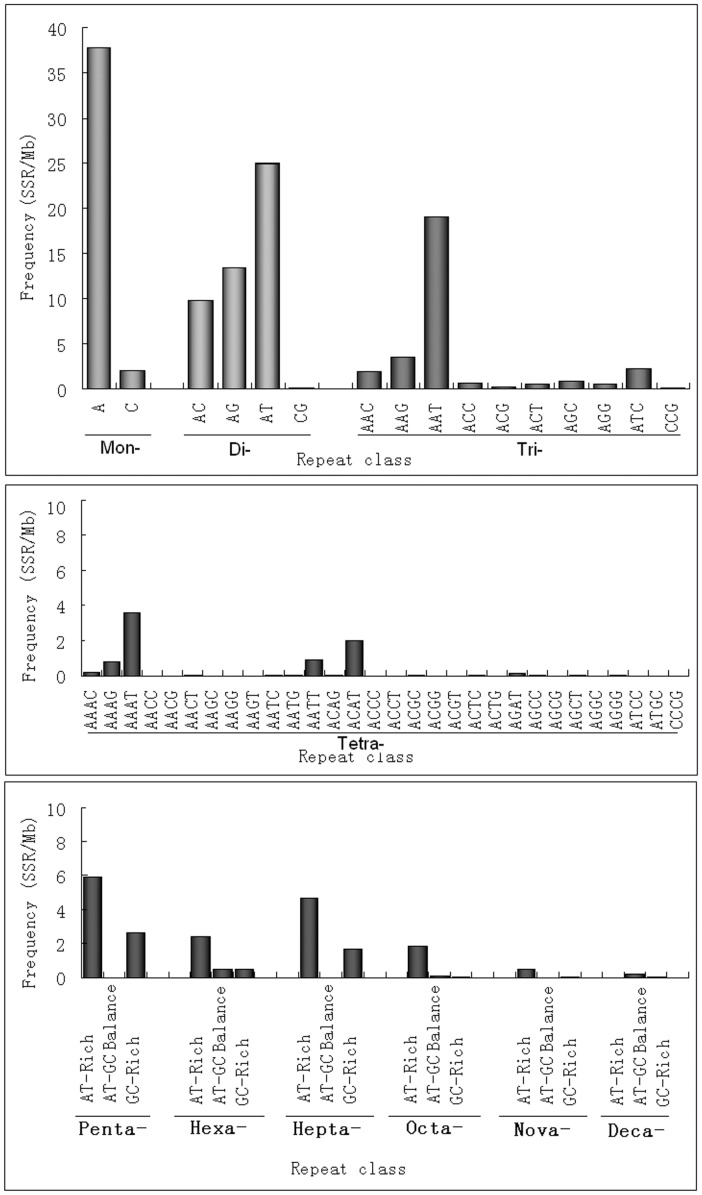
Frequencies of SSRs with certain patterns and base contents.

A more detailed investigation of individual repeat types was performed and presented in Figure 2 and Table S3 in File S1. As expected A/T repeats are more abundant than G/C repeats. The most frequent dinucleotide repeat unit is AT, while GC is very rare. AT dinucleotide repeats account for 16.99% of all SSRs, which reveals that AT repeats are the overall most common repeat type in sweet orange genome. AAT is the most frequent and CCG the least frequent trinucleotide repeat pattern. The predominant tetranucleotide repeat is AAAT, whereas GC-rich repeats like ACCC, AGGC, AGGG, CCCG are rare. AT-rich motifs are predominant in penta-, hexa- and heptanucleotide repeats, such as AAAAT, AAAAG, AAATT and AATAT, which are the most common pentanucleotide repeats. AAAAAG, AAAAAT and AAAAAC motifs are prevailed among hexanucleotides. Octa-, nona- and decanucleotides are underrepresented repeat types in the sweet orange genome, altogether accounting for only 1.95% of the total SSRs. AT-rich repeat patterns such as AAAAAAAG, AAAAAAAT and AAAAAATT are most frequent among the octanucleotide repeats, whereas AAAAAAAAG, AAAAAAAAT and AAAAAAAAC are most abundant among nonanucleotide repeats. The AACAATTATT and AAAAAAAAAG patterns are most abundant among decanucleotide repeats.

### Genome wide SSR marker development and in silico polymorphism analysis

**Figure 3:**
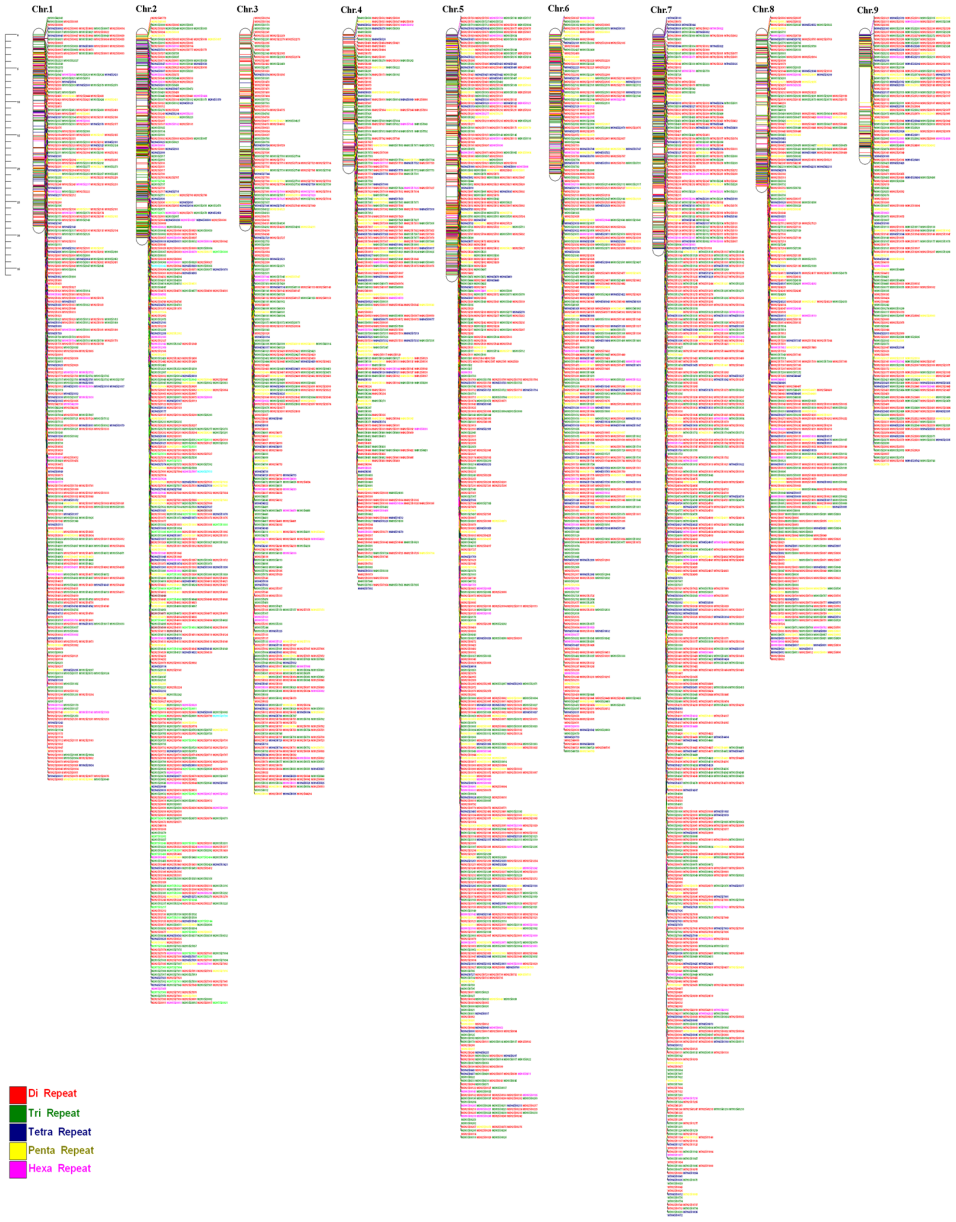
Chromosome maps depicting SSR loci of 5824 developed markers along the nine chromosomes of *C. sinensis*.

One of the primary goals of this study was to develop genome-wide SSR markers. Primers were designed for most of the di- to hexanucleotide repeats and results are presented in Table 3. A total of 21,248 SSR markers were successfully designed from the nine chromosomes. The distribution of SSR markers among the nine chromosomes was not significantly different. On average 2360 SSR markers were designed for each chromosome with 89 markers per Mb, covering the whole genome with gaps of less than 12 kb (Figure 3).

**Table 3:** Chromosome wide SSR marker development.

SSR repeat class	Chr1	Chr2	Chr3	Chr4	Chr5	Chr6	Chr7	Chr8	Chr9	Total
Dinucleotides	882	1403	1669	1546	1258	900	1296	848	765	10567
Trinucleotides	561	807	914	816	717	526	733	519	458	6051
Tetranucleotides	159	252	263	251	231	144	214	180	164	1858
Pentanucleotides	213	256	319	260	210	185	205	190	162	2000
Hexanucleotides	56	120	118	93	100	75	88	81	41	772
Total	1871	2838	3283	2966	2516	1830	2536	1818	1590	21248

**Figure 4:**
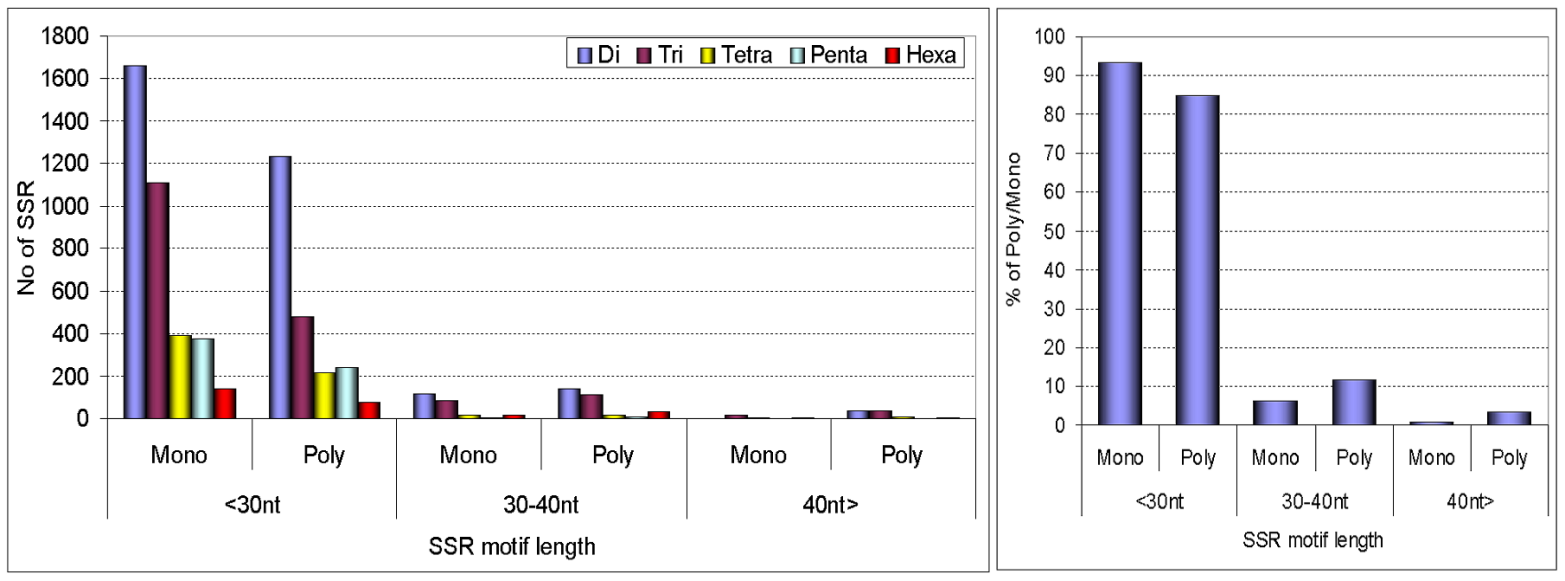
Frequency distribution of *C. sinensis* SSRs by repeat length, in monomorphic and polymorphic SSRs obtained from *in silico* PCR analyses.

Virtual PCR (VPCR) was performed for *in silico* polymorphism analysis and results are presented in Figure 4 and Table S4 in File S1. SSR markers are classified either as polymorphic or monomorphic based on the *in silico* amplicon size comparison. A total of 6588 (31%) *C. sinensis* primers amplified specific bands *in silico* from the genome of *C. clementina*. The remaining primers failed to generate specific amplicons or generated no amplicons. Among the 6588 SSR markers with amplicons, 3941 (59%) were polymorphic and 2647 (40%) were monomorphic as indicated in Figure 4. The relationship between degree of polymorphism and repeat length for each SSR type was estimated. We observe that shorter repeats are more monomorphic than long repeats, while polymorphic SSRs include a considerably higher percentage of long repeats. Dinucleotide repeats are more polymorphic than other repeat types and trinucleotide repeats have the highest proportion of monomorphic repeats.

### Functional annotation, wet lab validation of SSR markers and utility of newly developed GW-SSR resource for citrus research

**Figure 5:**
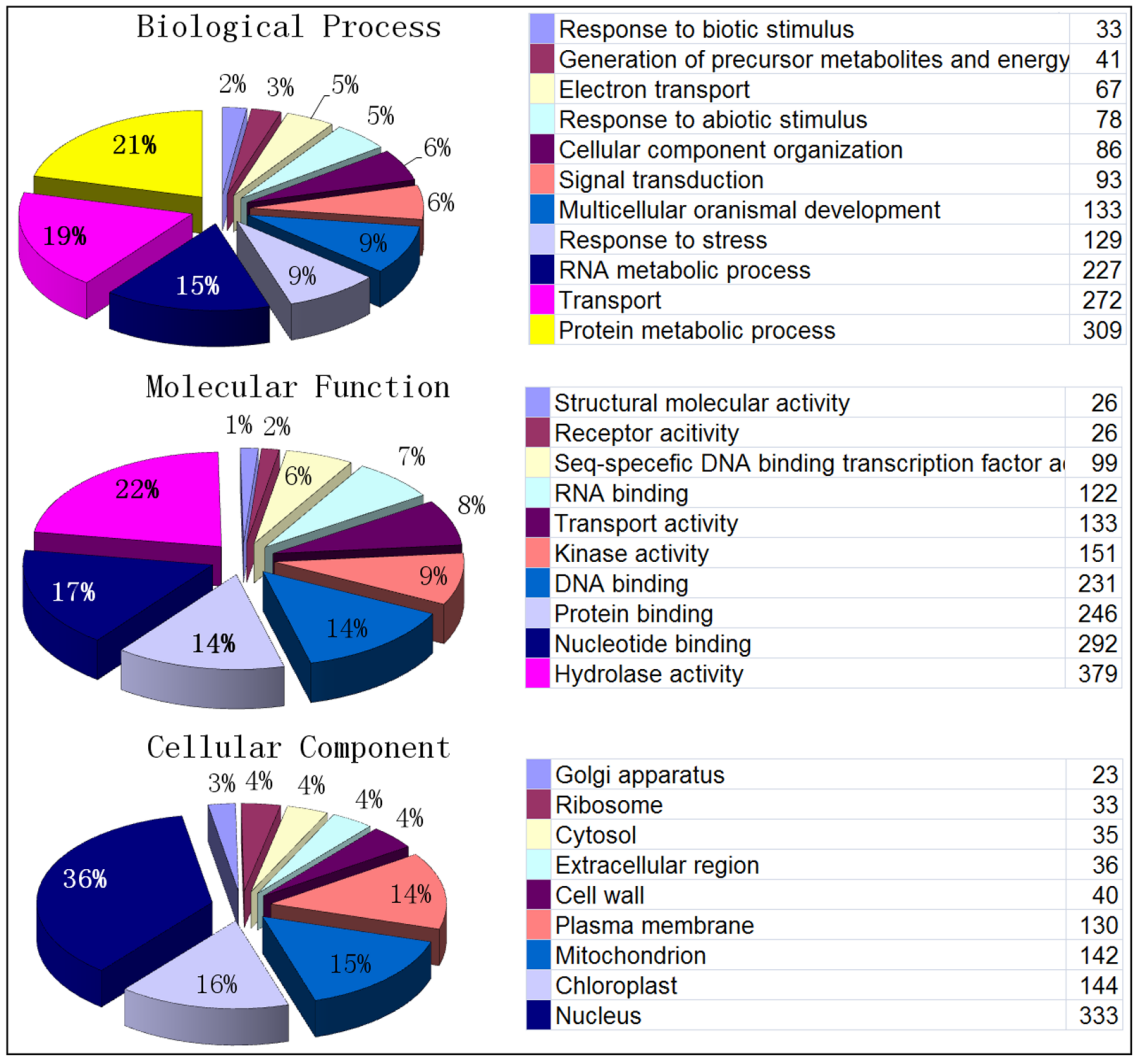
Gene Ontology annotation of genome wide developed sweet orange SSR marker flanking regions (A), the GO biological process, (B) molecular function and (C) cellular component.

Functional annotation of SSR loci was performed by a Blast2Go analysis. A significant GO annotation was found for 1870 loci, whereas 19378 loci had no significant homology to known sequences in the public databases (Figure 5). The majority of SSR loci for which an annotation was found are involved in protein metabolic processes (309), transport (272), and RNA metabolic process (227). When mapped against the molecular function category, 379 (20%), 292 (16%), 246 (13%) and 231 (12%) SSR loci were involved in hydrolase activity, nucleotide binding, protein binding and DNA binding, respectively. When mapped against the cellular component GO terms, 333 SSR loci (18%) were involved in nucleus and 144 (8%) were involved in chloroplast function.

**Figure 6:**
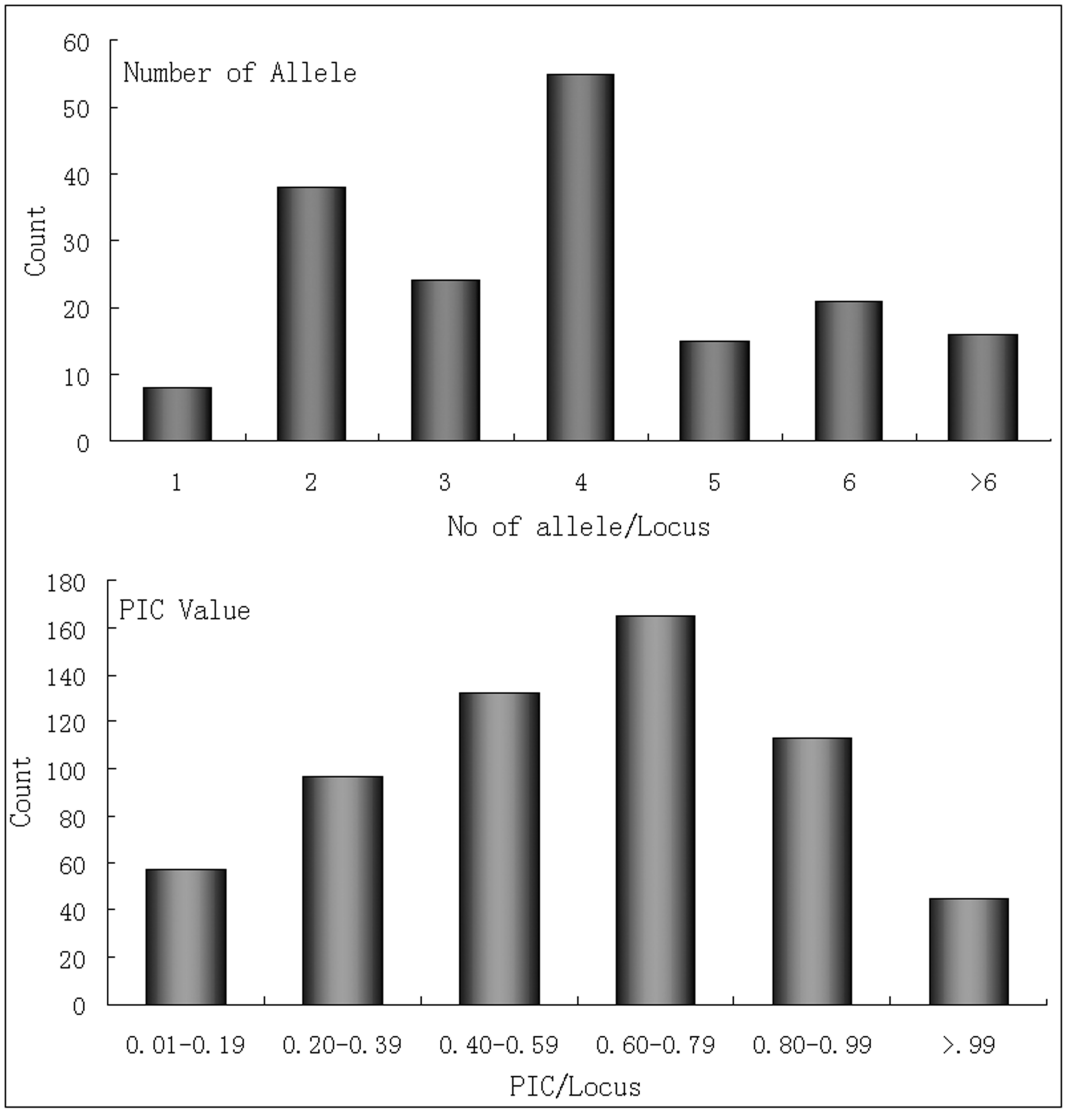
Number counts of allele frequencies and PIC values for the 609 SSRs analyzed in 16 Citrus germplasms.

A subset of 950 markers had been selected for wet lab experiments. Of these, 64.11% could be amplified in all 16 citrus accessions with prominent PCR products having the expected size. Among the tested primer pairs, 578 amplified in orange accessions, while 56.11%, 57.58% and 50.42% amplified in *C. grandis*, lemon (*C.limonia*) and trifoliata orange (*Poncirus trifoliata*), respectively. Highest transferability to the relative species was found in lemon followed by *C. grandis* and Kumquat (*Fortunella*) (Table S10 in File S1). A total of 2547 alleles were recorded from 609 SSR loci with an average of 4 alleles per loci. The majority of the primer amplified 4 alleles, followed by 2 and 3 respectively (Figure 6). The PIC value varied from 0.10 to 0.95 with an average of 0.73. The majority of the PIC values were found in the interval from 0.60 to 0.79 (Figure 6).

### Database of SSR marker and genome wide localization

A total of 21,248 SSR primer pairs were designed. For an easy access and utilization of these markers, we developed the sweet orange SSR Marker database, which stores the exact positions of these SSRs in the sweet orange genome assemble (Figure 3), the repeat pattern, expected PCR product length, primer temperature as well as the virtual PCR result are available in Table S11 in File S2.

## Discussion

### Frequency and distribution of SSRs in the sweet orange genome

We analyzed the genome-wide SSR distribution, frequency and density in the unit size range of 1 to 10 bp in *C. sinensis*. SSRs contributed 0.5% to the 347 Mbp genome assembly of *C. sinensis* and we found 1 SSR per 6.8 Kbp. This result is comparable with the SSR densities reported for other plant species [16], [17], [18]. In general, the frequency of SSRs is considerably higher in dicot species compare to monocots [4]. Our comparison of the SSR frequency in the *C. sinensis* and the *S. bicolor* genome shows a 2 fold higher frequency in the sweet orange genome (see Table S5, S6 in File S1). The majority of the SSRs were identified in chromosome 5, although the density of SSRs is the highest in chromosome 4. Many reports have demonstrated that SSRs are non-randomly distributed in genomes of various species [5], [19]. We performed a chi-square test to test for differences in the SSR distribution among different genomic regions and also the distribution among nine pseudo chromosomes (Table S7 in File S1). The chi square test supports a non-random distribution of SSRs in the sweet orange genome. We observed that the number of SSRs is negatively correlated with the genome size (r = −0.70). Similar observations have also been reported by Cavagnaro et al. [20]. Different SSR characteristics have been documented for different genomic regions in plant genomes. For example, Mun et al. [18] reported that SSR frequencies are higher in intergenic regions than in transcribed regions. In agreement with this observation, our results show a 1.5 fold higher SSR frequency in intergenic regions compared to transcribed regions of the sweet orange genome. Furthermore, we observed that among untranscribed regions, SSRs are densest in 5′-UTR, followed by introns and 3′-UTR regions. Similar observations for SSR densities in different genomic regions have been made for fungi [21], [22] and plants [4]. The presence of certain polymorphic SSRs in coding regions could modify the coding protein. SSRs in UTRs or introns could affect the level of gene expression, which could even lead to phenotypic changes. Li et al. [23] and Zhang et al. [24] demonstrated that variations in 3′-UTR or 5′-UTR SSRs could be responsible for regulating the translation of proteins and for mRNA stabilization. The SSR elements in 5′-UTR region are essential for some gene regulation adaptation as well as phenotypic changes on short time scales. In addition, evidence for SSRs in coding region which affect phenotypes in human MMR genes has been reported by Duval and Hamelin [25] as well as Vassileva et al. [26]. In this light, the high densities of SSRs in 5′-UTRs of the sweet orange genome should be seen as an opportunity to study the influence of SSRs on citrus gene regulation.

In sweet orange, most SSRs are mono-, di- and trinucleotide repeats, which together account for about 80% of all of the SSRs. We found that dinucleotide repeats are most frequent in the sweet orange genome, where trinucleotide repeats are most frequent in the cucumber [20] genome. This difference could arise from different search parameters used [20]. Cavagnaro et al. [20] explained how different SSR frequencies can be obtained in different studies due to differences in SSR search parameters and search algorithms. Therefore, in order to compare the frequency of SSRs in different plant species, the same program should be used with exactly the same search parameters. For this reason, we calculate the SSR frequencies of 11 plant genomes including citrus (see Table S5, S6 in File S1). Our data reveal that dinucleotide repeats are predominant in both monocot and dicot genomes and that the occurrence of different types of SSRs greatly vary with the search parameters. Surprisingly, heptanucleotide repeats show a higher density than hexanucleotide repeats in the sweet orange genome. Similar trends are also found in dicot species but not in monocot plant species. So it has been postulated that the decrease in SSR frequency with unit length is higher in monocot species as compared to dicot species. The distribution of microsatellites across the different genomic regions show that all repeat types except tri- and hexanucleotide repeats were comparatively less frequent in CDS regions compared to the other genomic region of the sweet orange genome. The high frequency of trinucleotide repeats in protein coding regions (CDS) has previously been reported for several plant species [16], [17], [18], [27], [28] and other eukaryotes including insects and human [29], [30], [31]. The predominance of tri- and hexanucleotide repeats in CDS regions can be explained by the fact that their mutations won't disrupt the reading frame. We also found evidence for positive selection for specific repeats in the sweet orange genome by comparing the repeat density of a specific patter with its reverse complement on the sense strand in transcribed regions. This effect is called a standedness [5]. For example, the CT pattern is over-represent in 5′-UTR but not 3′-UTR compared to the AG pattern (Table S8, S9 in File S1). It is believed that oligo pyrimidine tracts in the 5′-terminal may be involved in the regulation of translation in vertebrate mRNA and also known as the plant translational apparatus [32]. Evidences suggested that CT microsatellites in 5′UTR of *Arabidopsis thaliana* are involved in their antisense transcription [33].

As shown in Figure 1, there is a remarkable variation in the frequency of individual repeat patterns in the sweet orange genome. The base composition of sweet orange SSR patterns is strongly biased toward A and T for all unit sizes. To give an example, the density of mono repeats A/T was 20 fold higher than for G/C patterns. This result is consistent with the previous studies where AT rich repeats have been found to be characteristic for dicot plants but not for monocots. The prevalence of AT over GC rich repeats seems to correlate with the overall genome composition. Indeed, the GC content in dicot genomes is comparatively lower than in monocot genomes. High differences among repeat pattern densities have also been reported for example for the Brassicaceae where the GAA/TTC and AAG/CTT trinucleotide patterns were the most frequent [34], while Hong et al. [35] found AAG/CTT to be the most abundant pattern in *Arabidopsis* and *B. rapa*. In the Solanaceae, the GAA/TCC and AGA/TCT patterns are most frequent, while CCG/CGG are the most frequent patterns found in the Solanaceae family [35] and in the coccolithophore *Emiliania huxleyi* [36].

### Genome wide SSR marker development, in silico polymorphism analysis, Functional annotation and wet lab validation of SSR markers

As noted for example by Cavagnaro et al. [20] mononucleotide repeats are not suitable for marker development. Therefore, we only considered di- to hexanucleotides repeats for primer modeling. In this size range, 33855 SSRs were identified in the sweet orange genome, but only 21248 (62.76%) loci are suitable for SSR marker development. SSR loci can be unsuitable for marker development due to insufficiently flanking regions. *In silico* polymorphisms of the developed markers were estimated by using the virtual PCR strategy. That allowed us to understand the possible relationships between the degree of polymorphism and particular features of sweet orange microsatellites. It is known that long repeats are more prone to mutations, which shrink the repeat, both in plants and animals [20], [37], [38]. Similar trends are found in our study. Comparing *C. sinensis* and *C. clementina*, we found that most SSR alleles from *C. sinensis* showed a length reduction in the corresponding *C. clementina* alleles. This result could be biased by the selection of the SSR loci analyzed, or the differences in SSR mutation rates between the two genotypes. Usually larger repeats are selected during SSR marker development; increasing the chances for a biased selection.

As expected, the majority of the SSR markers had no GO assignment since most SSRs are located in the intergenic regions of the sweet orange genome. However, a total 9% of the SSR markers had significant Gene Ontology hits. SSR loci with GO terms are good candidates as molecular markers for association studies.

In total, 950 SSRs that had been evaluated *in silico* were confirmed by wet lab experiments (i.e. PCR and gel electrophoresis) and for most of them, the results are consistent with the virtual PCR result. We obtained a high PCR amplification efficiency in this study (609 primer yielded scorable amplicons) which is consistent with earlier studies of marker development in plant species [39]. As expected, we found that the degree of marker transferability is higher in intra-specific populations than in inter-specific populations. The high degree of intra and inter specific transferability of markers will have a broad utilization in taxonomic, population conservation as well as mapping studies. Finally, it will assist breeding programs of citrus relatives, especially for species for which only few markers have been developed to data (such as lemon, lime, citron and kumquat etc.). The transferability of sweet orange SSR markers across *Citrus* species is higher than reported for other plant species [40], [41]. In general, the transferability rate within genus and among the genus greatly varies with the phylogenetic distances of the examined species and the genomic region used for marker development. EST-derived SSRs are more conserved than genomic-SSRs, therefore EST-SSRs are more transferable to related genera than genomics SSRs.

## Conclusion

The present study contributes to a detailed characterization and utilization of genome wide SSR markers in sweet orange. The sweet orange genome has a prevalence for AT-rich SSRs and SSRs are non-randomly distributed. A large number of markers have been developed and almost two thirds of these are transferable as well as polymorphic among citrus relatives. The knowledge of these markers significantly contributes to enhance the genomic resources in citrus species and will facilitate a number of genetic and genomic studies in citrus, including genetic diversity evaluation, population genetics, high density linkage map, positional cloning, and comparative genomics in other citrus species.

## Supporting Information

Figure S1 **Relative frequency (%) of SSR types, by number of repeats in the sweet orange genome.**
(TIF)

File S1 **Supporting files. Table S1, List of plat material used in wet lab experiment. Table S2, Mean number of repeats units observed in sweet orange genome. Table S3, Detailed investigation of individual repeat types. Table S4,** ***In silico*** **PCR result with** ***C. clementina*** **. Table S5, Results of microsatellite search. Table S6, Distribution to different repeat type classes among the 11 plant species. Table S7, Chi-square and correlation analysis. Table S8, Strand specificity of perfect microsatellites in** ***C. sinensis*** **transcribed regions. Table S9, SSR distribution in different genomic fraction on sweet orange genome. Table S10, Summary of the wet lab experiment.**
(DOC)

File S2 **Supporting file. Table S11,** ***Citrus sinensis*** **SSR marker data base.**
(XLSX)
