# Supplementary figures and images for: Genome Wide Characterization of Short Tandem Repeat Markers in Sweet Orange (Citrus sinensis)
Source: PLoS One. 2014 Aug 22;9(8):e104182. doi: 10.1371/journal.pone.0104182 (PMC4141690; doi:10.1371/journal.pone.0104182)

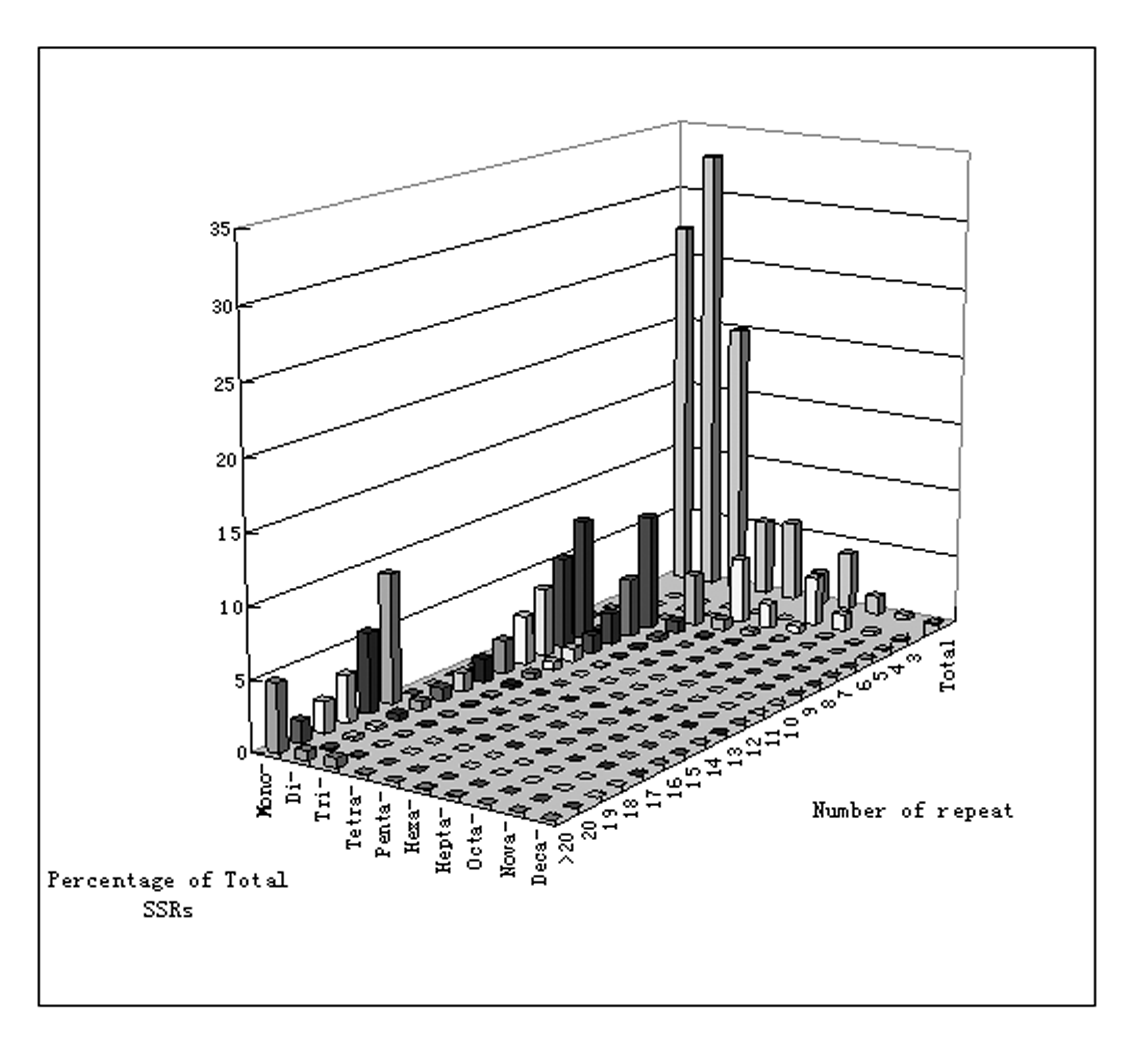

Supplement: Figure S1 — Relative frequency (%) of SSR types, by number of repeats in the sweet orange genome. (TIF) [file pone.0104182.s001.tif]
