# Supplementary material for: Genome Wide Characterization of Short Tandem Repeat Markers in Sweet Orange (Citrus sinensis)
Source: PLoS One. 2014 Aug 22;9(8):e104182. doi: 10.1371/journal.pone.0104182 (PMC4141690; doi:10.1371/journal.pone.0104182)
Supplement: File S1 — Supporting files. Table S1, List of plat material used in wet lab experiment. Table S2, Mean number of repeats units observed in sweet orange genome. Table S3, Detailed investigation of individual repeat types. Table S4, In silico PCR result with C. clementina . Table S5, Results of microsatellite search. Table S6, Distribution to different repeat type classes among the 11 plant species. Table S7, Chi-square and correlation analysis. Table S8, Strand specificity of perfect microsatellites in C. sinensis transcribed regions. Table S9, SSR distribution in different genomic fraction on sweet orange genome. Table S10, Summary of the wet lab experiment. (DOC) [file pone.0104182.s002.doc]

**Table S1 .** List of plat material Used in Wet lab Experiment.

| Sl No | Species name | *Scientific name* |
| --- | --- | --- |
| 1 | Valencia | *C. sinensis* |
| 2 | Newhall | *C. sinensis* |
| 3 | Mashuiju | *C. reticulata* |
| 4 | Hongju | *C. reticulata* |
| 5 | Eureka Lemon | *C. limon* |
| 6 | Mexican Lime | *C. aurantiifolia* |
| 7 | HB pummelo | *C. grandis* |
| 8 | Jinshi pummelo | *C. grandis* |
| 9 | Wendan pummelo | *C. grandis* |
| 10 | Kumquat 01 | *Fortunella sp* |
| 11 | Kumquat 03 | *Fortunella sp* |
| 12 | Kumquat (GZ01) | *Fortunella sp* |
| 13 | Kumquat (ZL01) | *Fortunella sp* |
| 14 | Kumquat (RYS01) | *Fortunella sp* |
| 15 | Trifoliata 1 | *Poncirus trifoliata* |
| 16 | Trifoliata 2 | *Poncirus trifoliata* |

**Table S2 .** Mean number of repeats units observed in Sweet orange genome.

|  | Mean number of repeat units | Minimum number of repeat units | Maximum number of repeat units |
| --- | --- | --- | --- |
| Mono- | 23.95 | 16 | 9758 |
| Di- | 14.39 | 8 | 6448 |
| Tri- | 16.12 | 6 | 6426 |
| Tetra- | 7.35 | 5 | 1831 |
| Penta- | 4.29 | 4 | 70 |
| Hexa- | 4.85 | 4 | 78 |
| Hepta- | 5.78 | 3 | 1135 |
| Octa- | 3.21 | 3 | 32 |
| Nova- | 3.08 | 3 | 4 |
| Deca- | 3.08 | 3 | 4 |

**Table S3 .** Detailed investigation of individual repeat types.

| Repeats | Total | Frequency (%) | Density perMb |
| --- | --- | --- | --- |
| A/T | 13124 | 25.811 | 37.792 |
| C/G | 664 | 1.306 | 1.912 |
| AC/GT | 3400 | 6.687 | 9.791 |
| AG/CT | 4650 | 9.145 | 13.390 |
| AT/AT | 8641 | 16.994 | 24.883 |
| CG/CG | 26 | 0.051 | 0.075 |
| AAC/GTT | 634 | 1.247 | 1.826 |
| AAG/CTT | 1221 | 2.401 | 3.516 |
| AAT/ATT | 6607 | 12.994 | 19.026 |
| ACC/GGT | 214 | 0.421 | 0.616 |
| ACG/CGT | 53 | 0.104 | 0.153 |
| ACT/AGT | 169 | 0.332 | 0.487 |
| AGC/CTG | 290 | 0.570 | 0.835 |
| AGG/CCT | 169 | 0.332 | 0.487 |
| ATC/ATG | 770 | 1.514 | 2.217 |
| CCG/CGG | 31 | 0.061 | 0.089 |
| AAAC/GTTT | 70 | 0.138 | 0.202 |
| AAAG/CTTT | 287 | 0.564 | 0.826 |
| AAAT/ATTT | 1248 | 2.454 | 3.594 |
| AACC/GGTT | 5 | 0.010 | 0.014 |
| AACG/CGTT | 3 | 0.006 | 0.009 |
| AACT/AGTT | 12 | 0.024 | 0.035 |
| AAGC/CTTG | 6 | 0.012 | 0.017 |
| AAGG/CCTT | 6 | 0.012 | 0.017 |
| AAGT/ACTT | 1 | 0.002 | 0.003 |
| AATC/ATTG | 25 | 0.049 | 0.072 |
| AATG/ATTC | 23 | 0.045 | 0.066 |
| AATT/AATT | 312 | 0.614 | 0.898 |
| ACAG/CTGT | 11 | 0.022 | 0.032 |
| ACAT/ATGT | 703 | 1.383 | 2.024 |
| ACCC/GGGT | 7 | 0.014 | 0.020 |
| ACCT/AGGT | 3 | 0.006 | 0.009 |
| ACGC/CGTG | 9 | 0.018 | 0.026 |
| ACGG/CCGT | 2 | 0.004 | 0.006 |
| ACGT/ACGT | 5 | 0.010 | 0.014 |
| ACTC/AGTG | 14 | 0.028 | 0.040 |
| ACTG/AGTC | 2 | 0.004 | 0.006 |
| AGAT/ATCT | 49 | 0.096 | 0.141 |
| AGCC/CTGG | 14 | 0.028 | 0.040 |
| AGCG/CGCT | 7 | 0.014 | 0.020 |
| AGCT/AGCT | 12 | 0.024 | 0.035 |
| AGGC/CCTG | 7 | 0.014 | 0.020 |
| AGGG/CCCT | 9 | 0.018 | 0.026 |
| ATCC/ATGG | 6 | 0.012 | 0.017 |
| ATGC/ATGC | 6 | 0.012 | 0.017 |
| CCCG/CGGG | 1 | 0.002 | 0.003 |
| AAAAC/GTTTT | 105 | 0.207 | 0.302 |
| AAAAG/CTTTT | 332 | 0.653 | 0.956 |
| AAAAT/ATTTT | 869 | 1.709 | 2.502 |
| AAACC/GGTTT | 26 | 0.051 | 0.075 |
| AAACG/CGTTT | 8 | 0.016 | 0.023 |
| AAACT/AGTTT | 17 | 0.033 | 0.049 |
| AAAGC/CTTTG | 14 | 0.028 | 0.040 |
| AAAGG/CCTTT | 18 | 0.035 | 0.052 |
| AAAGT/ACTTT | 3 | 0.006 | 0.009 |
| AAATC/ATTTG | 47 | 0.092 | 0.135 |
| AAATG/ATTTC | 10 | 0.020 | 0.029 |
| AAATT/AATTT | 193 | 0.380 | 0.556 |
| AACAC/GTGTT | 15 | 0.030 | 0.043 |
| AACAG/CTGTT | 5 | 0.010 | 0.014 |
| AACAT/ATGTT | 21 | 0.041 | 0.060 |
| AACCC/GGGTT | 22 | 0.043 | 0.063 |
| AACCG/CGGTT | 215 | 0.423 | 0.619 |
| AACCT/AGGTT | 3 | 0.006 | 0.009 |
| AACGC/CGTTG | 3 | 0.006 | 0.009 |
| AACGG/CCGTT | 2 | 0.004 | 0.006 |
| AACGT/ACGTT | 1 | 0.002 | 0.003 |
| AACTC/AGTTG | 4 | 0.008 | 0.012 |
| AACTG/AGTTC | 6 | 0.012 | 0.017 |
| AAGAC/CTTGT | 4 | 0.008 | 0.012 |
| AAGAG/CTCTT | 45 | 0.089 | 0.130 |
| AAGAT/ATCTT | 1 | 0.002 | 0.003 |
| AAGCC/CTTGG | 3 | 0.006 | 0.009 |
| AAGCT/AGCTT | 1 | 0.002 | 0.003 |
| AAGGC/CCTTG | 4 | 0.008 | 0.012 |
| AAGGG/CCCTT | 3 | 0.006 | 0.009 |
| AAGTC/ACTTG | 2 | 0.004 | 0.006 |
| AAGTG/ACTTC | 6 | 0.012 | 0.017 |
| AATAC/ATTGT | 8 | 0.016 | 0.023 |
| AATAG/ATTCT | 2 | 0.004 | 0.006 |
| AATAT/ATATT | 158 | 0.311 | 0.455 |
| AATCC/ATTGG | 40 | 0.079 | 0.115 |
| AATCG/ATTCG | 11 | 0.022 | 0.032 |
| AATCT/AGATT | 88 | 0.173 | 0.253 |
| AATGC/ATTGC | 1 | 0.002 | 0.003 |
| AATGT/ACATT | 2 | 0.004 | 0.006 |
| AATTC/AATTG | 18 | 0.035 | 0.052 |
| ACACC/GGTGT | 1 | 0.002 | 0.003 |
| ACACG/CGTGT | 3 | 0.006 | 0.009 |
| ACAGC/CTGTG | 5 | 0.010 | 0.014 |
| ACAGG/CCTGT | 1 | 0.002 | 0.003 |
| ACAGT/ACTGT | 1 | 0.002 | 0.003 |
| ACATC/ATGTG | 3 | 0.006 | 0.009 |
| ACATG/ATGTC | 4 | 0.008 | 0.012 |
| ACCAT/ATGGT | 1 | 0.002 | 0.003 |
| ACCCC/GGGGT | 22 | 0.043 | 0.063 |
| ACCCG/CGGGT | 31 | 0.061 | 0.089 |
| ACCCT/AGGGT | 1 | 0.002 | 0.003 |
| ACCGG/CCGGT | 2 | 0.004 | 0.006 |
| ACCTC/AGGTG | 3 | 0.006 | 0.009 |
| ACCTG/AGGTC | 1 | 0.002 | 0.003 |
| ACGAG/CGTCT | 6 | 0.012 | 0.017 |
| ACGAT/ATCGT | 2 | 0.004 | 0.006 |
| ACGCC/CGTGG | 2 | 0.004 | 0.006 |
| ACGGC/CCGTG | 23 | 0.045 | 0.066 |
| ACGTC/ACGTG | 1 | 0.002 | 0.003 |
| ACTAG/AGTCT | 2 | 0.004 | 0.006 |
| ACTAT/AGTAT | 2 | 0.004 | 0.006 |
| ACTCC/AGTGG | 5 | 0.010 | 0.014 |
| ACTCT/AGAGT | 1 | 0.002 | 0.003 |
| ACTGC/AGTGC | 3 | 0.006 | 0.009 |
| AGAGC/CTCTG | 2 | 0.004 | 0.006 |
| AGAGG/CCTCT | 23 | 0.045 | 0.066 |
| AGATC/ATCTG | 2 | 0.004 | 0.006 |
| AGATG/ATCTC | 13 | 0.026 | 0.037 |
| AGCAT/ATGCT | 1 | 0.002 | 0.003 |
| AGCCC/CTGGG | 13 | 0.026 | 0.037 |
| AGCCG/CGGCT | 3 | 0.006 | 0.009 |
| AGCCT/AGGCT | 1 | 0.002 | 0.003 |
| AGCTC/AGCTG | 8 | 0.016 | 0.023 |
| AGGCC/CCTGG | 1 | 0.002 | 0.003 |
| AGGGC/CCCTG | 1 | 0.002 | 0.003 |
| AGGGG/CCCCT | 369 | 0.726 | 1.063 |
| ATATC/ATATG | 8 | 0.016 | 0.023 |
| ATCCC/ATGGG | 7 | 0.014 | 0.020 |
| ATCCG/ATCGG | 30 | 0.059 | 0.086 |
| ATCGC/ATGCG | 3 | 0.006 | 0.009 |
| ATGCC/ATGGC | 3 | 0.006 | 0.009 |
| CCCCG/CGGGG | 1 | 0.002 | 0.003 |
| CCCGG/CCGGG | 3 | 0.006 | 0.009 |
| CCGCG/CGCGG | 1 | 0.002 | 0.003 |
| AAAAAC/GTTTTT | 56 | 0.110 | 0.161 |
| AAAAAG/CTTTTT | 144 | 0.283 | 0.415 |
| AAAAAT/ATTTTT | 190 | 0.374 | 0.547 |
| AAAACC/GGTTTT | 8 | 0.016 | 0.023 |
| AAAACG/CGTTTT | 1 | 0.002 | 0.003 |
| AAAACT/AGTTTT | 2 | 0.004 | 0.006 |
| AAAAGC/CTTTTG | 22 | 0.043 | 0.063 |
| AAAAGG/CCTTTT | 4 | 0.008 | 0.012 |
| AAAAGT/ACTTTT | 4 | 0.008 | 0.012 |
| AAAATC/ATTTTG | 11 | 0.022 | 0.032 |
| AAAATG/ATTTTC | 13 | 0.026 | 0.037 |
| AAAATT/AATTTT | 46 | 0.090 | 0.132 |
| AAACAC/GTGTTT | 7 | 0.014 | 0.020 |
| AAACAG/CTGTTT | 2 | 0.004 | 0.006 |
| AAACAT/ATGTTT | 1 | 0.002 | 0.003 |
| AAACCC/GGGTTT | 4 | 0.008 | 0.012 |
| AAACCG/CGGTTT | 1 | 0.002 | 0.003 |
| AAACGG/CCGTTT | 1 | 0.002 | 0.003 |
| AAACTC/AGTTTG | 1 | 0.002 | 0.003 |
| AAACTG/AGTTTC | 3 | 0.006 | 0.009 |
| AAAGAC/CTTTGT | 4 | 0.008 | 0.012 |
| AAAGAG/CTCTTT | 22 | 0.043 | 0.063 |
| AAAGAT/ATCTTT | 14 | 0.028 | 0.040 |
| AAAGCC/CTTTGG | 3 | 0.006 | 0.009 |
| AAAGCG/CGCTTT | 1 | 0.002 | 0.003 |
| AAAGGC/CCTTTG | 2 | 0.004 | 0.006 |
| AAAGGG/CCCTTT | 1 | 0.002 | 0.003 |
| AAAGTC/ACTTTG | 1 | 0.002 | 0.003 |
| AAAGTG/ACTTTC | 1 | 0.002 | 0.003 |
| AAATAC/ATTTGT | 3 | 0.006 | 0.009 |
| AAATAT/ATATTT | 14 | 0.028 | 0.040 |
| AAATCC/ATTTGG | 4 | 0.008 | 0.012 |
| AAATCG/ATTTCG | 3 | 0.006 | 0.009 |
| AAATCT/AGATTT | 3 | 0.006 | 0.009 |
| AAATGC/ATTTGC | 1 | 0.002 | 0.003 |
| AAATGG/ATTTCC | 4 | 0.008 | 0.012 |
| AAATGT/ACATTT | 1 | 0.002 | 0.003 |
| AAATTC/AATTTG | 4 | 0.008 | 0.012 |
| AAATTG/AATTTC | 1 | 0.002 | 0.003 |
| AAATTT/AAATTT | 4 | 0.008 | 0.012 |
| AACAAG/CTTGTT | 4 | 0.008 | 0.012 |
| AACAAT/ATTGTT | 5 | 0.010 | 0.014 |
| AACACC/GGTGTT | 3 | 0.006 | 0.009 |
| AACAGC/CTGTTG | 4 | 0.008 | 0.012 |
| AACAGG/CCTGTT | 1 | 0.002 | 0.003 |
| AACAGT/ACTGTT | 1 | 0.002 | 0.003 |
| AACATC/ATGTTG | 5 | 0.010 | 0.014 |
| AACATG/ATGTTC | 1 | 0.002 | 0.003 |
| AACCAG/CTGGTT | 5 | 0.010 | 0.014 |
| AACCCG/CGGGTT | 3 | 0.006 | 0.009 |
| AACCCT/AGGGTT | 6 | 0.012 | 0.017 |
| AACCTC/AGGTTG | 1 | 0.002 | 0.003 |
| AACCTG/AGGTTC | 1 | 0.002 | 0.003 |
| AACGCC/CGTTGG | 2 | 0.004 | 0.006 |
| AACGGC/CCGTTG | 2 | 0.004 | 0.006 |
| AACGGT/ACCGTT | 4 | 0.008 | 0.012 |
| AACTAC/AGTTGT | 2 | 0.004 | 0.006 |
| AACTAG/AGTTCT | 2 | 0.004 | 0.006 |
| AACTCC/AGTTGG | 1 | 0.002 | 0.003 |
| AACTCT/AGAGTT | 1 | 0.002 | 0.003 |
| AACTGG/AGTTCC | 1 | 0.002 | 0.003 |
| AACTGT/ACAGTT | 1 | 0.002 | 0.003 |
| AACTTC/AAGTTG | 4 | 0.008 | 0.012 |
| AACTTG/AAGTTC | 1 | 0.002 | 0.003 |
| AAGAAT/ATTCTT | 1 | 0.002 | 0.003 |
| AAGACC/CTTGGT | 1 | 0.002 | 0.003 |
| AAGACG/CGTCTT | 12 | 0.024 | 0.035 |
| AAGAGC/CTCTTG | 1 | 0.002 | 0.003 |
| AAGAGG/CCTCTT | 13 | 0.026 | 0.037 |
| AAGATC/ATCTTG | 2 | 0.004 | 0.006 |
| AAGATG/ATCTTC | 19 | 0.037 | 0.055 |
| AAGCAC/CTTGTG | 5 | 0.010 | 0.014 |
| AAGCAG/CTGCTT | 5 | 0.010 | 0.014 |
| AAGCAT/ATGCTT | 1 | 0.002 | 0.003 |
| AAGCCC/CTTGGG | 4 | 0.008 | 0.012 |
| AAGCTC/AGCTTG | 2 | 0.004 | 0.006 |
| AAGGAC/CCTTGT | 3 | 0.006 | 0.009 |
| AAGGAG/CCTTCT | 12 | 0.024 | 0.035 |
| AAGGAT/ATCCTT | 1 | 0.002 | 0.003 |
| AAGGCT/AGCCTT | 1 | 0.002 | 0.003 |
| AAGGGC/CCCTTG | 1 | 0.002 | 0.003 |
| AAGGGG/CCCCTT | 1 | 0.002 | 0.003 |
| AAGGTG/ACCTTC | 2 | 0.004 | 0.006 |
| AAGTCC/ACTTGG | 2 | 0.004 | 0.006 |
| AAGTGC/ACTTGC | 3 | 0.006 | 0.009 |
| AAGTGG/ACTTCC | 1 | 0.002 | 0.003 |
| AATACT/AGTATT | 2 | 0.004 | 0.006 |
| AATAGC/ATTGCT | 1 | 0.002 | 0.003 |
| AATAGT/ACTATT | 3 | 0.006 | 0.009 |
| AATATC/ATATTG | 4 | 0.008 | 0.012 |
| AATATG/ATATTC | 1 | 0.002 | 0.003 |
| AATATT/AATATT | 5 | 0.010 | 0.014 |
| AATCAC/ATTGTG | 2 | 0.004 | 0.006 |
| AATCAG/ATTCTG | 26 | 0.051 | 0.075 |
| AATCAT/ATGATT | 10 | 0.020 | 0.029 |
| AATCCC/ATTGGG | 2 | 0.004 | 0.006 |
| AATCCG/ATTCGG | 1 | 0.002 | 0.003 |
| AATCCT/AGGATT | 5 | 0.010 | 0.014 |
| AATCGC/ATTGCG | 1 | 0.002 | 0.003 |
| AATCGG/ATTCCG | 5 | 0.010 | 0.014 |
| AATCGT/ACGATT | 1 | 0.002 | 0.003 |
| AATCTC/AGATTG | 5 | 0.010 | 0.014 |
| AATCTG/AGATTC | 1 | 0.002 | 0.003 |
| AATGAC/ATTGTC | 1 | 0.002 | 0.003 |
| AATGAG/ATTCTC | 1 | 0.002 | 0.003 |
| AATGAT/ATCATT | 5 | 0.010 | 0.014 |
| AATGCC/ATTGGC | 2 | 0.004 | 0.006 |
| AATGCG/ATTCGC | 1 | 0.002 | 0.003 |
| AATGCT/AGCATT | 1 | 0.002 | 0.003 |
| AATGGC/ATTGCC | 4 | 0.008 | 0.012 |
| AATGGG/ATTCCC | 1 | 0.002 | 0.003 |
| AATGGT/ACCATT | 1 | 0.002 | 0.003 |
| AATGTG/ACATTC | 1 | 0.002 | 0.003 |
| AATTAG/AATTCT | 1 | 0.002 | 0.003 |
| AATTAT/AATTAT | 19 | 0.037 | 0.055 |
| AATTCC/AATTGG | 6 | 0.012 | 0.017 |
| ACACAG/CTGTGT | 1 | 0.002 | 0.003 |
| ACACAT/ATGTGT | 5 | 0.010 | 0.014 |
| ACACCC/GGGTGT | 2 | 0.004 | 0.006 |
| ACACCG/CGGTGT | 2 | 0.004 | 0.006 |
| ACACGC/CGTGTG | 5 | 0.010 | 0.014 |
| ACACTC/AGTGTG | 3 | 0.006 | 0.009 |
| ACACTG/AGTGTC | 1 | 0.002 | 0.003 |
| ACAGAG/CTCTGT | 7 | 0.014 | 0.020 |
| ACAGAT/ATCTGT | 1 | 0.002 | 0.003 |
| ACAGCC/CTGTGG | 7 | 0.014 | 0.020 |
| ACAGGC/CCTGTG | 1 | 0.002 | 0.003 |
| ACAGTC/ACTGTG | 3 | 0.006 | 0.009 |
| ACATAT/ATATGT | 44 | 0.087 | 0.127 |
| ACATCC/ATGTGG | 1 | 0.002 | 0.003 |
| ACATGC/ATGTGC | 1 | 0.002 | 0.003 |
| ACCACG/CGTGGT | 1 | 0.002 | 0.003 |
| ACCACT/AGTGGT | 2 | 0.004 | 0.006 |
| ACCAGC/CTGGTG | 6 | 0.012 | 0.017 |
| ACCAGG/CCTGGT | 1 | 0.002 | 0.003 |
| ACCATC/ATGGTG | 13 | 0.026 | 0.037 |
| ACCATG/ATGGTC | 1 | 0.002 | 0.003 |
| ACCCAG/CTGGGT | 1 | 0.002 | 0.003 |
| ACCCCC/GGGGGT | 1 | 0.002 | 0.003 |
| ACCCGG/CCGGGT | 10 | 0.020 | 0.029 |
| ACCCTC/AGGGTG | 14 | 0.028 | 0.040 |
| ACCCTG/AGGGTC | 1 | 0.002 | 0.003 |
| ACCGAG/CGGTCT | 1 | 0.002 | 0.003 |
| ACCGCC/CGGTGG | 1 | 0.002 | 0.003 |
| ACCGGC/CCGGTG | 2 | 0.004 | 0.006 |
| ACCGTC/ACGGTG | 2 | 0.004 | 0.006 |
| ACCTCC/AGGTGG | 7 | 0.014 | 0.020 |
| ACGACT/AGTCGT | 1 | 0.002 | 0.003 |
| ACGAGC/CGTGCT | 4 | 0.008 | 0.012 |
| ACGATC/ATCGTG | 1 | 0.002 | 0.003 |
| ACGATG/ATCGTC | 5 | 0.010 | 0.014 |
| ACGCCG/CGGCGT | 1 | 0.002 | 0.003 |
| ACGCTC/AGCGTG | 1 | 0.002 | 0.003 |
| ACGGAG/CCGTCT | 3 | 0.006 | 0.009 |
| ACGGCC/CCGTGG | 1 | 0.002 | 0.003 |
| ACGGCG/CCGTCG | 1 | 0.002 | 0.003 |
| ACGGGC/CCCGTG | 2 | 0.004 | 0.006 |
| ACGGGG/CCCCGT | 1 | 0.002 | 0.003 |
| ACGTAT/ACGTAT | 1 | 0.002 | 0.003 |
| ACTATC/AGTGAT | 3 | 0.006 | 0.009 |
| ACTCCC/AGTGGG | 14 | 0.028 | 0.040 |
| ACTCCG/AGTCGG | 1 | 0.002 | 0.003 |
| ACTCGC/AGTGCG | 3 | 0.006 | 0.009 |
| ACTCGG/AGTCCG | 5 | 0.010 | 0.014 |
| ACTCTC/AGAGTG | 5 | 0.010 | 0.014 |
| ACTGCC/AGTGGC | 1 | 0.002 | 0.003 |
| ACTGCG/AGTCGC | 1 | 0.002 | 0.003 |
| ACTGCT/AGCAGT | 1 | 0.002 | 0.003 |
| ACTGGG/AGTCCC | 1 | 0.002 | 0.003 |
| AGAGCC/CTCTGG | 1 | 0.002 | 0.003 |
| AGAGCG/CGCTCT | 1 | 0.002 | 0.003 |
| AGAGCT/AGCTCT | 2 | 0.004 | 0.006 |
| AGAGGC/CCTCTG | 3 | 0.006 | 0.009 |
| AGAGGG/CCCTCT | 17 | 0.033 | 0.049 |
| AGATAT/ATATCT | 28 | 0.055 | 0.081 |
| AGATCC/ATCTGG | 2 | 0.004 | 0.006 |
| AGATGG/ATCTCC | 4 | 0.008 | 0.012 |
| AGCAGG/CCTGCT | 1 | 0.002 | 0.003 |
| AGCATC/ATGCTG | 3 | 0.006 | 0.009 |
| AGCCAT/ATGGCT | 1 | 0.002 | 0.003 |
| AGCCCC/CTGGGG | 3 | 0.006 | 0.009 |
| AGCCGC/CGGCTG | 3 | 0.006 | 0.009 |
| AGCCGG/CCGGCT | 2 | 0.004 | 0.006 |
| AGCCTC/AGGCTG | 4 | 0.008 | 0.012 |
| AGCCTG/AGGCTC | 3 | 0.006 | 0.009 |
| AGCGCC/CGCTGG | 1 | 0.002 | 0.003 |
| AGCGCG/CGCGCT | 1 | 0.002 | 0.003 |
| AGCGGC/CCGCTG | 1 | 0.002 | 0.003 |
| AGCGGG/CCCGCT | 3 | 0.006 | 0.009 |
| AGCTCC/AGCTGG | 3 | 0.006 | 0.009 |
| AGCTGC/AGCTGC | 1 | 0.002 | 0.003 |
| AGGATG/ATCCTC | 4 | 0.008 | 0.012 |
| AGGCAT/ATGCCT | 1 | 0.002 | 0.003 |
| AGGCGG/CCGCCT | 1 | 0.002 | 0.003 |
| AGGGGC/CCCCTG | 1 | 0.002 | 0.003 |
| ATATCC/ATATGG | 3 | 0.006 | 0.009 |
| ATCATG/ATCATG | 1 | 0.002 | 0.003 |
| ATCCCC/ATGGGG | 5 | 0.010 | 0.014 |
| ATCCGG/ATCCGG | 4 | 0.008 | 0.012 |
| ATCGCC/ATGGCG | 2 | 0.004 | 0.006 |
| ATCGGC/ATGCCG | 1 | 0.002 | 0.003 |
| ATGCCC/ATGGGC | 2 | 0.004 | 0.006 |
| AAAAAAC/GTTTTTT | 42 | 0.083 | 0.121 |
| AAAAAAG/CTTTTTT | 329 | 0.647 | 0.947 |
| AAAAAAT/ATTTTTT | 477 | 0.938 | 1.374 |
| AAAAACC/GGTTTTT | 3 | 0.006 | 0.009 |
| AAAAACG/CGTTTTT | 2 | 0.004 | 0.006 |
| AAAAACT/AGTTTTT | 4 | 0.008 | 0.012 |
| AAAAAGC/CTTTTTG | 2 | 0.004 | 0.006 |
| AAAAAGG/CCTTTTT | 20 | 0.039 | 0.058 |
| AAAAAGT/ACTTTTT | 8 | 0.016 | 0.023 |
| AAAAATC/ATTTTTG | 10 | 0.020 | 0.029 |
| AAAAATG/ATTTTTC | 16 | 0.031 | 0.046 |
| AAAAATT/AATTTTT | 118 | 0.232 | 0.340 |
| AAAACAC/GTGTTTT | 3 | 0.006 | 0.009 |
| AAAACAG/CTGTTTT | 2 | 0.004 | 0.006 |
| AAAACAT/ATGTTTT | 13 | 0.026 | 0.037 |
| AAAACCC/GGGTTTT | 6 | 0.012 | 0.017 |
| AAAACCG/CGGTTTT | 1 | 0.002 | 0.003 |
| AAAACGC/CGTTTTG | 1 | 0.002 | 0.003 |
| AAAACTC/AGTTTTG | 2 | 0.004 | 0.006 |
| AAAACTG/AGTTTTC | 2 | 0.004 | 0.006 |
| AAAACTT/AAGTTTT | 6 | 0.012 | 0.017 |
| AAAAGAG/CTCTTTT | 1 | 0.002 | 0.003 |
| AAAAGAT/ATCTTTT | 2 | 0.004 | 0.006 |
| AAAAGCC/CTTTTGG | 12 | 0.024 | 0.035 |
| AAAAGCT/AGCTTTT | 1 | 0.002 | 0.003 |
| AAAAGGG/CCCTTTT | 4 | 0.008 | 0.012 |
| AAAAGGT/ACCTTTT | 1 | 0.002 | 0.003 |
| AAAAGTC/ACTTTTG | 2 | 0.004 | 0.006 |
| AAAAGTG/ACTTTTC | 6 | 0.012 | 0.017 |
| AAAAGTT/AACTTTT | 2 | 0.004 | 0.006 |
| AAAATAC/ATTTTGT | 7 | 0.014 | 0.020 |
| AAAATAG/ATTTTCT | 5 | 0.010 | 0.014 |
| AAAATAT/ATATTTT | 30 | 0.059 | 0.086 |
| AAAATCC/ATTTTGG | 2 | 0.004 | 0.006 |
| AAAATCG/ATTTTCG | 2 | 0.004 | 0.006 |
| AAAATCT/AGATTTT | 2 | 0.004 | 0.006 |
| AAAATGG/ATTTTCC | 6 | 0.012 | 0.017 |
| AAAATGT/ACATTTT | 4 | 0.008 | 0.012 |
| AAAATTC/AATTTTG | 20 | 0.039 | 0.058 |
| AAAATTG/AATTTTC | 7 | 0.014 | 0.020 |
| AAAATTT/AAATTTT | 47 | 0.092 | 0.135 |
| AAACAAC/GTTGTTT | 4 | 0.008 | 0.012 |
| AAACAAG/CTTGTTT | 1 | 0.002 | 0.003 |
| AAACAAT/ATTGTTT | 1 | 0.002 | 0.003 |
| AAACACT/AGTGTTT | 1 | 0.002 | 0.003 |
| AAACAGC/CTGTTTG | 1 | 0.002 | 0.003 |
| AAACAGT/ACTGTTT | 1 | 0.002 | 0.003 |
| AAACATG/ATGTTTC | 3 | 0.006 | 0.009 |
| AAACATT/AATGTTT | 2 | 0.004 | 0.006 |
| AAACCAC/GGTTTGT | 1 | 0.002 | 0.003 |
| AAACCAG/CTGGTTT | 1 | 0.002 | 0.003 |
| AAACCCC/GGGGTTT | 4 | 0.008 | 0.012 |
| AAACCCG/CGGGTTT | 4 | 0.008 | 0.012 |
| AAACCCT/AGGGTTT | 105 | 0.207 | 0.302 |
| AAACCGC/CGGTTTG | 1 | 0.002 | 0.003 |
| AAACCTG/AGGTTTC | 1 | 0.002 | 0.003 |
| AAACGGC/CCGTTTG | 1 | 0.002 | 0.003 |
| AAACGGG/CCCGTTT | 1 | 0.002 | 0.003 |
| AAACTAC/AGTTTGT | 1 | 0.002 | 0.003 |
| AAACTCC/AGTTTGG | 2 | 0.004 | 0.006 |
| AAACTGC/AGTTTGC | 3 | 0.006 | 0.009 |
| AAACTGG/AGTTTCC | 1 | 0.002 | 0.003 |
| AAACTTC/AAGTTTG | 2 | 0.004 | 0.006 |
| AAACTTG/AAGTTTC | 7 | 0.014 | 0.020 |
| AAAGAAC/CTTTGTT | 4 | 0.008 | 0.012 |
| AAAGAAG/CTTCTTT | 19 | 0.037 | 0.055 |
| AAAGAAT/ATTCTTT | 8 | 0.016 | 0.023 |
| AAAGACT/AGTCTTT | 1 | 0.002 | 0.003 |
| AAAGAGG/CCTCTTT | 4 | 0.008 | 0.012 |
| AAAGATC/ATCTTTG | 1 | 0.002 | 0.003 |
| AAAGATG/ATCTTTC | 3 | 0.006 | 0.009 |
| AAAGATT/AATCTTT | 3 | 0.006 | 0.009 |
| AAAGCAG/CTGCTTT | 1 | 0.002 | 0.003 |
| AAAGCAT/ATGCTTT | 3 | 0.006 | 0.009 |
| AAAGCCC/CTTTGGG | 2 | 0.004 | 0.006 |
| AAAGCCT/AGGCTTT | 3 | 0.006 | 0.009 |
| AAAGCTC/AGCTTTG | 1 | 0.002 | 0.003 |
| AAAGGAG/CCTTTCT | 3 | 0.006 | 0.009 |
| AAAGGAT/ATCCTTT | 1 | 0.002 | 0.003 |
| AAAGGCT/AGCCTTT | 1 | 0.002 | 0.003 |
| AAAGGGC/CCCTTTG | 1 | 0.002 | 0.003 |
| AAAGGTC/ACCTTTG | 1 | 0.002 | 0.003 |
| AAAGTCC/ACTTTGG | 2 | 0.004 | 0.006 |
| AAAGTCT/ACTTTAG | 1 | 0.002 | 0.003 |
| AAAGTGG/ACTTTCC | 2 | 0.004 | 0.006 |
| AAAGTTC/AACTTTG | 8 | 0.016 | 0.023 |
| AAATAAC/ATTTGTT | 6 | 0.012 | 0.017 |
| AAATAAG/ATTTCTT | 2 | 0.004 | 0.006 |
| AAATAAT/ATTATTT | 42 | 0.083 | 0.121 |
| AAATACC/ATTTGGT | 4 | 0.008 | 0.012 |
| AAATAGT/ACTATTT | 2 | 0.004 | 0.006 |
| AAATATC/ATATTTG | 3 | 0.006 | 0.009 |
| AAATATG/ATATTTC | 3 | 0.006 | 0.009 |
| AAATATT/AATATTT | 25 | 0.049 | 0.072 |
| AAATCAC/ATTTGTG | 4 | 0.008 | 0.012 |
| AAATCAG/ATTTCTG | 2 | 0.004 | 0.006 |
| AAATCAT/ATGATTT | 4 | 0.008 | 0.012 |
| AAATCTC/AGATTTG | 3 | 0.006 | 0.009 |
| AAATCTG/AGATTTC | 3 | 0.006 | 0.009 |
| AAATGAG/ATTTCTC | 1 | 0.002 | 0.003 |
| AAATGCC/ATTTGGC | 3 | 0.006 | 0.009 |
| AAATGCT/AGCATTT | 1 | 0.002 | 0.003 |
| AAATGGC/ATTTGCC | 3 | 0.006 | 0.009 |
| AAATGGG/ATTTCCC | 2 | 0.004 | 0.006 |
| AAATGTC/ACATTTG | 2 | 0.004 | 0.006 |
| AAATGTG/ACATTTC | 2 | 0.004 | 0.006 |
| AAATGTT/AACATTT | 2 | 0.004 | 0.006 |
| AAATTAC/AATTTGT | 2 | 0.004 | 0.006 |
| AAATTAG/AATTTCT | 3 | 0.006 | 0.009 |
| AAATTAT/AATTTAT | 16 | 0.031 | 0.046 |
| AAATTCC/AATTTGG | 2 | 0.004 | 0.006 |
| AAATTCT/AATTTAG | 1 | 0.002 | 0.003 |
| AAATTGG/AATTTCC | 6 | 0.012 | 0.017 |
| AAATTGT/AATTTAC | 4 | 0.008 | 0.012 |
| AAATTTC/AAATTTG | 9 | 0.018 | 0.026 |
| AACAACC/GGTTGTT | 1 | 0.002 | 0.003 |
| AACAACG/CGTTGTT | 2 | 0.004 | 0.006 |
| AACAATC/ATTGTTG | 5 | 0.010 | 0.014 |
| AACAATT/AATTGTT | 8 | 0.016 | 0.023 |
| AACACAC/GTGTGTT | 13 | 0.026 | 0.037 |
| AACACAG/CTGTGTT | 2 | 0.004 | 0.006 |
| AACACAT/ATGTGTT | 5 | 0.010 | 0.014 |
| AACACTC/AGTGTTG | 2 | 0.004 | 0.006 |
| AACACTG/AGTGTTC | 1 | 0.002 | 0.003 |
| AACACTT/AAGTGTT | 1 | 0.002 | 0.003 |
| AACAGAT/ATCTGTT | 1 | 0.002 | 0.003 |
| AACAGCT/AGCTGTT | 4 | 0.008 | 0.012 |
| AACAGGC/CCTGTTG | 2 | 0.004 | 0.006 |
| AACAGTC/ACTGTTG | 1 | 0.002 | 0.003 |
| AACAGTT/AACTGTT | 1 | 0.002 | 0.003 |
| AACATAC/ATGTTGT | 1 | 0.002 | 0.003 |
| AACATAT/ATATGTT | 2 | 0.004 | 0.006 |
| AACATGC/ATGTTGC | 2 | 0.004 | 0.006 |
| AACATGG/ATGTTCC | 1 | 0.002 | 0.003 |
| AACATTG/AATGTTC | 1 | 0.002 | 0.003 |
| AACCAAG/CTTGGTT | 6 | 0.012 | 0.017 |
| AACCAGC/CTGGTTG | 2 | 0.004 | 0.006 |
| AACCAGG/CCTGGTT | 2 | 0.004 | 0.006 |
| AACCCCT/AGGGGTT | 4 | 0.008 | 0.012 |
| AACCCGG/CCGGGTT | 3 | 0.006 | 0.009 |
| AACCCTC/AGGGTTG | 3 | 0.006 | 0.009 |
| AACCCTG/AGGGTTC | 1 | 0.002 | 0.003 |
| AACCGTC/ACGGTTG | 2 | 0.004 | 0.006 |
| AACCTTC/AAGGTTG | 2 | 0.004 | 0.006 |
| AACGAGC/CGTTGCT | 1 | 0.002 | 0.003 |
| AACGCCC/CGTTGGG | 1 | 0.002 | 0.003 |
| AACGGGC/CCCGTTG | 1 | 0.002 | 0.003 |
| AACGGTC/ACCGTTG | 12 | 0.024 | 0.035 |
| AACGTGC/ACGTTGC | 1 | 0.002 | 0.003 |
| AACTAAG/AGTTCTT | 1 | 0.002 | 0.003 |
| AACTACT/AGTAGTT | 7 | 0.014 | 0.020 |
| AACTCAC/AGTTGTG | 2 | 0.004 | 0.006 |
| AACTCCC/AGTTGGG | 1 | 0.002 | 0.003 |
| AACTCGC/AGTTGCG | 1 | 0.002 | 0.003 |
| AACTCTC/AGAGTTG | 1 | 0.002 | 0.003 |
| AACTGAC/AGTTGTC | 2 | 0.004 | 0.006 |
| AACTGAG/AGTTCTC | 3 | 0.006 | 0.009 |
| AACTGCC/AGTTGGC | 1 | 0.002 | 0.003 |
| AACTGCT/AGCAGTT | 1 | 0.002 | 0.003 |
| AACTGGG/AGTTCCC | 1 | 0.002 | 0.003 |
| AACTGTC/ACAGTTG | 1 | 0.002 | 0.003 |
| AACTTGC/AAGTTGC | 8 | 0.016 | 0.023 |
| AAGAAGC/CTTCTTG | 2 | 0.004 | 0.006 |
| AAGAATC/ATTCTTG | 2 | 0.004 | 0.006 |
| AAGAATG/ATTCTTC | 6 | 0.012 | 0.017 |
| AAGACAG/CTGTCTT | 1 | 0.002 | 0.003 |
| AAGACGC/CGTCTTG | 1 | 0.002 | 0.003 |
| AAGACTC/AGTCTTG | 2 | 0.004 | 0.006 |
| AAGACTG/AGTCTTC | 1 | 0.002 | 0.003 |
| AAGACTT/AAGTCTT | 2 | 0.004 | 0.006 |
| AAGAGAG/CTCTCTT | 10 | 0.020 | 0.029 |
| AAGAGCC/CTCTTGG | 2 | 0.004 | 0.006 |
| AAGAGGC/CCTCTTG | 1 | 0.002 | 0.003 |
| AAGAGGG/CCCTCTT | 2 | 0.004 | 0.006 |
| AAGAGGT/ACCTCTT | 1 | 0.002 | 0.003 |
| AAGAGTG/ACTCTTC | 1 | 0.002 | 0.003 |
| AAGATAC/ATCTTGT | 1 | 0.002 | 0.003 |
| AAGATAG/ATCTTCT | 2 | 0.004 | 0.006 |
| AAGATCC/ATCTTGG | 1 | 0.002 | 0.003 |
| AAGATCT/AGATCTT | 2 | 0.004 | 0.006 |
| AAGATTC/AATCTTG | 5 | 0.010 | 0.014 |
| AAGATTG/AATCTTC | 2 | 0.004 | 0.006 |
| AAGCACC/CTTGGTG | 2 | 0.004 | 0.006 |
| AAGCAGG/CCTGCTT | 1 | 0.002 | 0.003 |
| AAGCATC/ATGCTTG | 3 | 0.006 | 0.009 |
| AAGCATG/ATGCTTC | 2 | 0.004 | 0.006 |
| AAGCCAG/CTGGCTT | 1 | 0.002 | 0.003 |
| AAGCCAT/ATGGCTT | 2 | 0.004 | 0.006 |
| AAGCCCC/CTTGGGG | 1 | 0.002 | 0.003 |
| AAGCCCG/CGGGCTT | 1 | 0.002 | 0.003 |
| AAGCCCT/AGGGCTT | 3 | 0.006 | 0.009 |
| AAGCCTG/AGGCTTC | 1 | 0.002 | 0.003 |
| AAGCCTT/AAGGCTT | 1 | 0.002 | 0.003 |
| AAGCGGC/CCGCTTG | 1 | 0.002 | 0.003 |
| AAGCGGG/CCCGCTT | 1 | 0.002 | 0.003 |
| AAGCTGC/AGCTTGC | 1 | 0.002 | 0.003 |
| AAGCTGG/AGCTTCC | 1 | 0.002 | 0.003 |
| AAGCTTC/AAGCTTG | 4 | 0.008 | 0.012 |
| AAGGAAT/ATTCCTT | 2 | 0.004 | 0.006 |
| AAGGACG/CCTTCGT | 1 | 0.002 | 0.003 |
| AAGGATC/ATCCTTG | 1 | 0.002 | 0.003 |
| AAGGCAC/CCTTGTG | 1 | 0.002 | 0.003 |
| AAGGCCT/AGGCCTT | 1 | 0.002 | 0.003 |
| AAGGCGG/CCGCCTT | 3 | 0.006 | 0.009 |
| AAGGCTC/AGCCTTG | 3 | 0.006 | 0.009 |
| AAGGCTG/AGCCTTC | 1 | 0.002 | 0.003 |
| AAGGGTC/ACCCTTG | 1 | 0.002 | 0.003 |
| AAGGGTG/ACCCTTC | 2 | 0.004 | 0.006 |
| AAGGTAT/ACCTTAT | 1 | 0.002 | 0.003 |
| AAGGTCG/ACCTTCG | 1 | 0.002 | 0.003 |
| AAGTCAC/ACTTGTG | 2 | 0.004 | 0.006 |
| AAGTCGT/ACGACTT | 1 | 0.002 | 0.003 |
| AAGTGAG/ACTTCTC | 3 | 0.006 | 0.009 |
| AAGTGCC/ACTTGGC | 1 | 0.002 | 0.003 |
| AAGTGGC/ACTTGCC | 6 | 0.012 | 0.017 |
| AAGTGGT/ACCACTT | 2 | 0.004 | 0.006 |
| AAGTGTG/ACACTTC | 1 | 0.002 | 0.003 |
| AATAATC/ATTATTG | 3 | 0.006 | 0.009 |
| AATAATG/ATTATTC | 2 | 0.004 | 0.006 |
| AATAATT/AATTATT | 18 | 0.035 | 0.052 |
| AATACAG/ATTCTGT | 2 | 0.004 | 0.006 |
| AATACCC/ATTGGGT | 2 | 0.004 | 0.006 |
| AATACTG/AGTATTC | 3 | 0.006 | 0.009 |
| AATAGCC/ATTGGCT | 1 | 0.002 | 0.003 |
| AATAGCT/AGCTATT | 1 | 0.002 | 0.003 |
| AATAGTG/ACTATTC | 1 | 0.002 | 0.003 |
| AATATAT/ATATATT | 5 | 0.010 | 0.014 |
| AATATCT/AGATATT | 1 | 0.002 | 0.003 |
| AATATGC/ATATTGC | 3 | 0.006 | 0.009 |
| AATCACC/ATTGGTG | 3 | 0.006 | 0.009 |
| AATCACG/ATTCGTG | 1 | 0.002 | 0.003 |
| AATCACT/AGTGATT | 1 | 0.002 | 0.003 |
| AATCAGC/ATTGCTG | 1 | 0.002 | 0.003 |
| AATCATC/ATGATTG | 2 | 0.004 | 0.006 |
| AATCATG/ATGATTC | 3 | 0.006 | 0.009 |
| AATCATT/AATGATT | 1 | 0.002 | 0.003 |
| AATCCAG/ATTCTGG | 2 | 0.004 | 0.006 |
| AATCCCC/ATTGGGG | 1 | 0.002 | 0.003 |
| AATCCTC/AGGATTG | 3 | 0.006 | 0.009 |
| AATCCTG/AGGATTC | 1 | 0.002 | 0.003 |
| AATCGGC/ATTGCCG | 1 | 0.002 | 0.003 |
| AATCTCC/AGATTGG | 2 | 0.004 | 0.006 |
| AATGACC/ATTGGTC | 1 | 0.002 | 0.003 |
| AATGAGG/ATTCCTC | 3 | 0.006 | 0.009 |
| AATGAGT/ACTCATT | 1 | 0.002 | 0.003 |
| AATGATG/ATCATTC | 33 | 0.065 | 0.095 |
| AATGCAT/ATGCATT | 2 | 0.004 | 0.006 |
| AATGCCC/ATTGGGC | 1 | 0.002 | 0.003 |
| AATGCCG/ATTCGGC | 1 | 0.002 | 0.003 |
| AATGCCT/AGGCATT | 1 | 0.002 | 0.003 |
| AATGCGG/ATTCCGC | 7 | 0.014 | 0.020 |
| AATGCTC/AGCATTG | 3 | 0.006 | 0.009 |
| AATGGAC/ATTGTCC | 1 | 0.002 | 0.003 |
| AATGGAG/ATTCTCC | 4 | 0.008 | 0.012 |
| AATGGCC/ATTGGCC | 1 | 0.002 | 0.003 |
| AATGGCG/ATTCGCC | 1 | 0.002 | 0.003 |
| AATGGGC/ATTGCCC | 1 | 0.002 | 0.003 |
| AATGGGG/ATTCCCC | 3 | 0.006 | 0.009 |
| AATGGTG/ACCATTC | 1 | 0.002 | 0.003 |
| AATGTAC/ACATTGT | 1 | 0.002 | 0.003 |
| AATGTAT/ACATTAT | 2 | 0.004 | 0.006 |
| AATGTGG/ACATTCC | 2 | 0.004 | 0.006 |
| AATGTGT/ACACATT | 1 | 0.002 | 0.003 |
| AATTACC/AATTGGT | 1 | 0.002 | 0.003 |
| AATTACT/AATTAGT | 2 | 0.004 | 0.006 |
| AATTAGC/AATTGCT | 3 | 0.006 | 0.009 |
| AATTATC/AATTGAT | 5 | 0.010 | 0.014 |
| AATTATG/AATTCAT | 6 | 0.012 | 0.017 |
| AATTCTC/AATTGAG | 1 | 0.002 | 0.003 |
| AATTGCC/AATTGGC | 2 | 0.004 | 0.006 |
| ACACACC/GGTGTGT | 3 | 0.006 | 0.009 |
| ACACCCT/AGGGTGT | 3 | 0.006 | 0.009 |
| ACACCGC/CGGTGTG | 2 | 0.004 | 0.006 |
| ACACCTC/AGGTGTG | 1 | 0.002 | 0.003 |
| ACACGGC/CCGTGTG | 1 | 0.002 | 0.003 |
| ACACGTG/ACGTGTC | 1 | 0.002 | 0.003 |
| ACACTCC/AGTGTGG | 16 | 0.031 | 0.046 |
| ACACTGG/AGTGTCC | 3 | 0.006 | 0.009 |
| ACAGACG/CGTCTGT | 1 | 0.002 | 0.003 |
| ACAGCCC/CTGTGGG | 1 | 0.002 | 0.003 |
| ACAGCCT/AGGCTGT | 2 | 0.004 | 0.006 |
| ACAGCGG/CCGCTGT | 1 | 0.002 | 0.003 |
| ACAGCTG/AGCTGTC | 1 | 0.002 | 0.003 |
| ACAGGGT/ACCCTGT | 1 | 0.002 | 0.003 |
| ACAGGTG/ACCTGTC | 1 | 0.002 | 0.003 |
| ACAGTAG/ACTGTCT | 1 | 0.002 | 0.003 |
| ACAGTCT/ACTGTAG | 1 | 0.002 | 0.003 |
| ACAGTGG/ACTGTCC | 1 | 0.002 | 0.003 |
| ACATATC/ATATGTG | 1 | 0.002 | 0.003 |
| ACATCTC/AGATGTG | 1 | 0.002 | 0.003 |
| ACATGAT/ATCATGT | 2 | 0.004 | 0.006 |
| ACATGCT/AGCATGT | 1 | 0.002 | 0.003 |
| ACATGTC/ACATGTG | 1 | 0.002 | 0.003 |
| ACCACCC/GGGTGGT | 2 | 0.004 | 0.006 |
| ACCACCG/CGGTGGT | 5 | 0.010 | 0.014 |
| ACCACCT/AGGTGGT | 3 | 0.006 | 0.009 |
| ACCAGAG/CTCTGGT | 2 | 0.004 | 0.006 |
| ACCAGCC/CTGGTGG | 4 | 0.008 | 0.012 |
| ACCATGC/ATGGTGC | 2 | 0.004 | 0.006 |
| ACCCAGC/CTGGGTG | 1 | 0.002 | 0.003 |
| ACCCAGG/CCTGGGT | 1 | 0.002 | 0.003 |
| ACCCATC/ATGGGTG | 1 | 0.002 | 0.003 |
| ACCCATG/ATGGGTC | 2 | 0.004 | 0.006 |
| ACCCCCC/GGGGGGT | 1 | 0.002 | 0.003 |
| ACCCGTC/ACGGGTG | 1 | 0.002 | 0.003 |
| ACCCTAG/AGGGTCT | 1 | 0.002 | 0.003 |
| ACCCTAT/AGGGTAT | 4 | 0.008 | 0.012 |
| ACCGAGT/ACTCGGT | 1 | 0.002 | 0.003 |
| ACCGTAG/ACGGTCT | 1 | 0.002 | 0.003 |
| ACCTCTG/AGAGGTC | 1 | 0.002 | 0.003 |
| ACCTGCG/AGGTCGC | 1 | 0.002 | 0.003 |
| ACGATCT/AGATCGT | 4 | 0.008 | 0.012 |
| ACGGAGT/ACTCCGT | 1 | 0.002 | 0.003 |
| ACGGCAG/CCGTCTG | 2 | 0.004 | 0.006 |
| ACGGCTC/AGCCGTG | 6 | 0.012 | 0.017 |
| ACGGGCC/CCCGTGG | 1 | 0.002 | 0.003 |
| ACGTCCC/ACGTGGG | 1 | 0.002 | 0.003 |
| ACGTCTC/ACGTGAG | 1 | 0.002 | 0.003 |
| ACGTGCC/ACGTGGC | 1 | 0.002 | 0.003 |
| ACTAGAG/AGTCTCT | 1 | 0.002 | 0.003 |
| ACTAGGC/AGTGCCT | 1 | 0.002 | 0.003 |
| ACTATGG/AGTCCAT | 1 | 0.002 | 0.003 |
| ACTCAGT/ACTGAGT | 1 | 0.002 | 0.003 |
| ACTCATC/AGTGATG | 1 | 0.002 | 0.003 |
| ACTCCAG/AGTCTGG | 2 | 0.004 | 0.006 |
| ACTCCAT/AGTATGG | 1 | 0.002 | 0.003 |
| ACTCCCC/AGTGGGG | 7 | 0.014 | 0.020 |
| ACTCCCT/AGGGAGT | 1 | 0.002 | 0.003 |
| ACTCCTC/AGGAGTG | 4 | 0.008 | 0.012 |
| ACTCCTG/AGGAGTC | 1 | 0.002 | 0.003 |
| ACTCGGC/AGTGCCG | 2 | 0.004 | 0.006 |
| ACTCTCC/AGAGTGG | 1 | 0.002 | 0.003 |
| ACTCTCG/AGAGTCG | 2 | 0.004 | 0.006 |
| ACTGATG/AGTCATC | 2 | 0.004 | 0.006 |
| ACTGCAG/AGTCTGC | 2 | 0.004 | 0.006 |
| ACTGCCC/AGTGGGC | 1 | 0.002 | 0.003 |
| ACTGCTC/AGCAGTG | 2 | 0.004 | 0.006 |
| ACTGGCG/AGTCGCC | 1 | 0.002 | 0.003 |
| AGAGAGC/CTCTCTG | 1 | 0.002 | 0.003 |
| AGAGAGG/CCTCTCT | 1 | 0.002 | 0.003 |
| AGAGATG/ATCTCTC | 3 | 0.006 | 0.009 |
| AGAGCCT/AGGCTCT | 1 | 0.002 | 0.003 |
| AGAGGCT/AGCCTCT | 1 | 0.002 | 0.003 |
| AGAGGGG/CCCCTCT | 1 | 0.002 | 0.003 |
| AGATAGG/ATCTCCT | 1 | 0.002 | 0.003 |
| AGATATG/ATATCTC | 9 | 0.018 | 0.026 |
| AGATGAT/ATCATCT | 1 | 0.002 | 0.003 |
| AGCAGCC/CTGCTGG | 3 | 0.006 | 0.009 |
| AGCAGCT/AGCTGCT | 1 | 0.002 | 0.003 |
| AGCAGGC/CCTGCTG | 2 | 0.004 | 0.006 |
| AGCATGC/ATGCTGC | 3 | 0.006 | 0.009 |
| AGCATGG/ATGCTCC | 11 | 0.022 | 0.032 |
| AGCCATC/ATGGCTG | 1 | 0.002 | 0.003 |
| AGCCATG/ATGGCTC | 2 | 0.004 | 0.006 |
| AGCCCAT/ATGGGCT | 1 | 0.002 | 0.003 |
| AGCCCCT/AGGGGCT | 1 | 0.002 | 0.003 |
| AGCCTGC/AGGCTGC | 1 | 0.002 | 0.003 |
| AGCGGCC/CCGCTGG | 1 | 0.002 | 0.003 |
| AGCGGGC/CCCGCTG | 1 | 0.002 | 0.003 |
| AGGAGGC/CCTCCTG | 1 | 0.002 | 0.003 |
| AGGAGGG/CCCTCCT | 1 | 0.002 | 0.003 |
| AGGGCGC/CCCTGCG | 1 | 0.002 | 0.003 |
| AGGGGGG/CCCCCCT | 1 | 0.002 | 0.003 |
| ATCATCC/ATGATGG | 2 | 0.004 | 0.006 |
| ATCATGC/ATGATGC | 1 | 0.002 | 0.003 |
| ATCCCCG/ATCGGGG | 1 | 0.002 | 0.003 |
| ATGCCCC/ATGGGGC | 1 | 0.002 | 0.003 |
| CCCCCGG/CCGGGGG | 1 | 0.002 | 0.003 |
| CCCCGGG/CCCGGGG | 1 | 0.002 | 0.003 |
| AAAAAAAC/GTTTTTTT | 24 | 0.047 | 0.069 |
| AAAAAAAG/CTTTTTTT | 97 | 0.191 | 0.279 |
| AAAAAAAT/ATTTTTTT | 99 | 0.195 | 0.285 |
| AAAAAAGC/CTTTTTTG | 4 | 0.008 | 0.012 |
| AAAAAAGG/CCTTTTTT | 3 | 0.006 | 0.009 |
| AAAAAAGT/ACTTTTTT | 1 | 0.002 | 0.003 |
| AAAAAATC/ATTTTTTG | 1 | 0.002 | 0.003 |
| AAAAAATG/ATTTTTTC | 1 | 0.002 | 0.003 |
| AAAAAATT/AATTTTTT | 43 | 0.085 | 0.124 |
| AAAAACAT/ATGTTTTT | 1 | 0.002 | 0.003 |
| AAAAACTT/AAGTTTTT | 2 | 0.004 | 0.006 |
| AAAAAGAG/CTCTTTTT | 1 | 0.002 | 0.003 |
| AAAAAGAT/ATCTTTTT | 1 | 0.002 | 0.003 |
| AAAAAGGC/CCTTTTTG | 1 | 0.002 | 0.003 |
| AAAAAGTG/ACTTTTTC | 1 | 0.002 | 0.003 |
| AAAAATAC/ATTTTTGT | 2 | 0.004 | 0.006 |
| AAAAATAG/ATTTTTCT | 3 | 0.006 | 0.009 |
| AAAAATAT/ATATTTTT | 10 | 0.020 | 0.029 |
| AAAAATCC/ATTTTTGG | 2 | 0.004 | 0.006 |
| AAAAATGG/ATTTTTCC | 2 | 0.004 | 0.006 |
| AAAAATTC/AATTTTTG | 10 | 0.020 | 0.029 |
| AAAAATTG/AATTTTTC | 2 | 0.004 | 0.006 |
| AAAAATTT/AAATTTTT | 5 | 0.010 | 0.014 |
| AAAACAAG/CTTGTTTT | 3 | 0.006 | 0.009 |
| AAAACAGC/CTGTTTTG | 2 | 0.004 | 0.006 |
| AAAACATT/AATGTTTT | 1 | 0.002 | 0.003 |
| AAAACCCC/GGGGTTTT | 1 | 0.002 | 0.003 |
| AAAACCCT/AGGGTTTT | 6 | 0.012 | 0.017 |
| AAAACTTC/AAGTTTTG | 1 | 0.002 | 0.003 |
| AAAAGAAC/CTTTTGTT | 2 | 0.004 | 0.006 |
| AAAAGAAG/CTTCTTTT | 2 | 0.004 | 0.006 |
| AAAAGAAT/ATTCTTTT | 4 | 0.008 | 0.012 |
| AAAAGAGC/CTCTTTTG | 1 | 0.002 | 0.003 |
| AAAAGAGG/CCTCTTTT | 1 | 0.002 | 0.003 |
| AAAAGCTG/AGCTTTTC | 1 | 0.002 | 0.003 |
| AAAAGTTT/AAACTTTT | 1 | 0.002 | 0.003 |
| AAAATAAG/ATTTTCTT | 1 | 0.002 | 0.003 |
| AAAATAAT/ATTATTTT | 11 | 0.022 | 0.032 |
| AAAATAGT/ACTATTTT | 1 | 0.002 | 0.003 |
| AAAATATG/ATATTTTC | 2 | 0.004 | 0.006 |
| AAAATATT/AATATTTT | 8 | 0.016 | 0.023 |
| AAAATCAT/ATGATTTT | 17 | 0.033 | 0.049 |
| AAAATCTC/AGATTTTG | 1 | 0.002 | 0.003 |
| AAAATGAC/ATTTTGTC | 1 | 0.002 | 0.003 |
| AAAATGAT/ATCATTTT | 1 | 0.002 | 0.003 |
| AAAATGTG/ACATTTTC | 2 | 0.004 | 0.006 |
| AAAATTAC/AATTTTGT | 3 | 0.006 | 0.009 |
| AAAATTAG/AATTTTCT | 3 | 0.006 | 0.009 |
| AAAATTAT/AATTTTAT | 11 | 0.022 | 0.032 |
| AAAATTCT/AATTTTAG | 2 | 0.004 | 0.006 |
| AAAATTGC/AATTTTGC | 1 | 0.002 | 0.003 |
| AAAATTTC/AAATTTTG | 2 | 0.004 | 0.006 |
| AAAATTTG/AAATTTTC | 4 | 0.008 | 0.012 |
| AAAATTTT/AAAATTTT | 10 | 0.020 | 0.029 |
| AAACAAAG/CTTTGTTT | 2 | 0.004 | 0.006 |
| AAACAAAT/ATTTGTTT | 2 | 0.004 | 0.006 |
| AAACAATC/ATTGTTTG | 2 | 0.004 | 0.006 |
| AAACACAC/GTGTGTTT | 1 | 0.002 | 0.003 |
| AAACAGAG/CTCTGTTT | 1 | 0.002 | 0.003 |
| AAACAGTC/ACTGTTTG | 2 | 0.004 | 0.006 |
| AAACCAAG/CTTGGTTT | 1 | 0.002 | 0.003 |
| AAACCATG/ATGGTTTC | 1 | 0.002 | 0.003 |
| AAACCGGG/CCCGGTTT | 1 | 0.002 | 0.003 |
| AAACGAAG/CGTTTCTT | 4 | 0.008 | 0.012 |
| AAACGAAT/ATTCGTTT | 1 | 0.002 | 0.003 |
| AAACTAAT/AGTTTATT | 1 | 0.002 | 0.003 |
| AAACTATC/AGTTTGAT | 2 | 0.004 | 0.006 |
| AAACTATT/AATAGTTT | 1 | 0.002 | 0.003 |
| AAACTCTT/AAGAGTTT | 1 | 0.002 | 0.003 |
| AAACTTAG/AAGTTTCT | 1 | 0.002 | 0.003 |
| AAAGAAGG/CCTTCTTT | 1 | 0.002 | 0.003 |
| AAAGAATT/AATTCTTT | 1 | 0.002 | 0.003 |
| AAAGACCG/CGGTCTTT | 1 | 0.002 | 0.003 |
| AAAGAGAG/CTCTCTTT | 14 | 0.028 | 0.040 |
| AAAGAGAT/ATCTCTTT | 2 | 0.004 | 0.006 |
| AAAGAGTC/ACTCTTTG | 1 | 0.002 | 0.003 |
| AAAGAGTG/ACTCTTTC | 1 | 0.002 | 0.003 |
| AAAGCAAT/ATTGCTTT | 1 | 0.002 | 0.003 |
| AAAGCAGC/CTGCTTTG | 1 | 0.002 | 0.003 |
| AAAGGAGG/CCTCCTTT | 2 | 0.004 | 0.006 |
| AAAGTAAT/ACTTTATT | 2 | 0.004 | 0.006 |
| AAAGTATG/ACTTTCAT | 1 | 0.002 | 0.003 |
| AAAGTCTC/ACTTTGAG | 2 | 0.004 | 0.006 |
| AAAGTCTG/ACTTTCAG | 1 | 0.002 | 0.003 |
| AAAGTGAG/ACTTTCTC | 1 | 0.002 | 0.003 |
| AAATAATC/ATTATTTG | 1 | 0.002 | 0.003 |
| AAATAATT/AATTATTT | 7 | 0.014 | 0.020 |
| AAATACCC/ATTTGGGT | 1 | 0.002 | 0.003 |
| AAATACTC/AGTATTTG | 1 | 0.002 | 0.003 |
| AAATAGAG/ATTTCTCT | 1 | 0.002 | 0.003 |
| AAATAGAT/ATCTATTT | 1 | 0.002 | 0.003 |
| AAATATAC/ATATTTGT | 6 | 0.012 | 0.017 |
| AAATATAT/ATATATTT | 5 | 0.010 | 0.014 |
| AAATATTC/AATATTTG | 2 | 0.004 | 0.006 |
| AAATATTT/AAATATTT | 1 | 0.002 | 0.003 |
| AAATCAAT/ATTGATTT | 1 | 0.002 | 0.003 |
| AAATCAGT/ACTGATTT | 1 | 0.002 | 0.003 |
| AAATCCTT/AAGGATTT | 1 | 0.002 | 0.003 |
| AAATCTTC/AAGATTTG | 1 | 0.002 | 0.003 |
| AAATGAAC/ATTTGTTC | 1 | 0.002 | 0.003 |
| AAATGAAG/ATTTCTTC | 1 | 0.002 | 0.003 |
| AAATGAGC/ATTTGCTC | 1 | 0.002 | 0.003 |
| AAATGAGT/ACTCATTT | 1 | 0.002 | 0.003 |
| AAATGTAG/ACATTTCT | 1 | 0.002 | 0.003 |
| AAATTAAT/AATTTATT | 6 | 0.012 | 0.017 |
| AAATTACG/AATTTCGT | 1 | 0.002 | 0.003 |
| AAATTATT/AATAATTT | 2 | 0.004 | 0.006 |
| AAATTCAG/AATTTCTG | 1 | 0.002 | 0.003 |
| AAATTCAT/AATTTATG | 1 | 0.002 | 0.003 |
| AAATTCTG/AATTTCAG | 1 | 0.002 | 0.003 |
| AAATTGAC/AATTTGTC | 1 | 0.002 | 0.003 |
| AAATTGCC/AATTTGGC | 1 | 0.002 | 0.003 |
| AAATTGCG/AATTTCGC | 1 | 0.002 | 0.003 |
| AAATTGTT/AACAATTT | 2 | 0.004 | 0.006 |
| AACAAGCC/CTTGTTGG | 1 | 0.002 | 0.003 |
| AACAAGGC/CCTTGTTG | 1 | 0.002 | 0.003 |
| AACAAGTC/ACTTGTTG | 1 | 0.002 | 0.003 |
| AACAATTC/AATTGTTG | 1 | 0.002 | 0.003 |
| AACACCCT/AGGGTGTT | 2 | 0.004 | 0.006 |
| AACACCTC/AGGTGTTG | 1 | 0.002 | 0.003 |
| AACAGACT/AGTCTGTT | 1 | 0.002 | 0.003 |
| AACATAAT/ATGTTATT | 1 | 0.002 | 0.003 |
| AACATATC/ATATGTTG | 2 | 0.004 | 0.006 |
| AACATCAT/ATGATGTT | 4 | 0.008 | 0.012 |
| AACATGCT/AGCATGTT | 1 | 0.002 | 0.003 |
| AACATGGC/ATGTTGCC | 1 | 0.002 | 0.003 |
| AACATTGT/AATGTTAC | 2 | 0.004 | 0.006 |
| AACCAATC/ATTGGTTG | 2 | 0.004 | 0.006 |
| AACCAGAT/ATCTGGTT | 1 | 0.002 | 0.003 |
| AACGCCCT/AGGGCGTT | 1 | 0.002 | 0.003 |
| AACGGGGG/CCCCCGTT | 1 | 0.002 | 0.003 |
| AACTATTC/AATAGTTG | 1 | 0.002 | 0.003 |
| AACTCATC/AGTTGATG | 1 | 0.002 | 0.003 |
| AACTCTTG/AAGAGTTC | 1 | 0.002 | 0.003 |
| AACTTAAT/AAGTTATT | 2 | 0.004 | 0.006 |
| AACTTCCT/AAGTTAGG | 1 | 0.002 | 0.003 |
| AACTTGAC/AAGTTGTC | 1 | 0.002 | 0.003 |
| AACTTGTC/AAGTTGAC | 1 | 0.002 | 0.003 |
| AAGAAGCC/CTTCTTGG | 1 | 0.002 | 0.003 |
| AAGAATTG/AATTCTTC | 5 | 0.010 | 0.014 |
| AAGACTAC/AGTCTTGT | 1 | 0.002 | 0.003 |
| AAGACTAG/AGTCTTCT | 1 | 0.002 | 0.003 |
| AAGAGAAT/ATTCTCTT | 1 | 0.002 | 0.003 |
| AAGAGATG/ATCTCTTC | 1 | 0.002 | 0.003 |
| AAGAGCAG/CTCTTCTG | 1 | 0.002 | 0.003 |
| AAGCATAC/ATGCTTGT | 1 | 0.002 | 0.003 |
| AAGCATGG/ATGCTTCC | 1 | 0.002 | 0.003 |
| AAGCCGAG/CGGCTTCT | 1 | 0.002 | 0.003 |
| AAGGAATG/ATTCCTTC | 1 | 0.002 | 0.003 |
| AAGGCGGG/CCCGCCTT | 1 | 0.002 | 0.003 |
| AAGTCATT/AATGACTT | 1 | 0.002 | 0.003 |
| AAGTCCTC/ACTTGAGG | 1 | 0.002 | 0.003 |
| AAGTGAGC/ACTTGCTC | 1 | 0.002 | 0.003 |
| AAGTGCAC/ACTTGTGC | 1 | 0.002 | 0.003 |
| AAGTGCAT/ACTTATGC | 2 | 0.004 | 0.006 |
| AATAATAC/ATTATTGT | 1 | 0.002 | 0.003 |
| AATAATAT/ATATTATT | 5 | 0.010 | 0.014 |
| AATAATGT/ACATTATT | 1 | 0.002 | 0.003 |
| AATAATTG/AATTATTC | 1 | 0.002 | 0.003 |
| AATATATT/AATATATT | 2 | 0.004 | 0.006 |
| AATATGAG/ATATTCTC | 2 | 0.004 | 0.006 |
| AATATTAC/AATATTGT | 2 | 0.004 | 0.006 |
| AATCACTG/AGTGATTC | 1 | 0.002 | 0.003 |
| AATCAGGG/ATTCCCTG | 1 | 0.002 | 0.003 |
| AATCATAC/ATGATTGT | 1 | 0.002 | 0.003 |
| AATCTCGC/AGATTGCG | 1 | 0.002 | 0.003 |
| AATGAATT/AATTCATT | 1 | 0.002 | 0.003 |
| AATGAGAG/ATTCTCTC | 2 | 0.004 | 0.006 |
| AATGAGTG/ACTCATTC | 1 | 0.002 | 0.003 |
| AATGGAGT/ACTCCATT | 1 | 0.002 | 0.003 |
| AATGTCCG/ACATTCGG | 1 | 0.002 | 0.003 |
| AATGTCTG/ACATTCAG | 2 | 0.004 | 0.006 |
| AATGTGAG/ACATTCTC | 1 | 0.002 | 0.003 |
| AATGTGGC/ACATTGCC | 1 | 0.002 | 0.003 |
| AATTACAC/AATTGTGT | 2 | 0.004 | 0.006 |
| AATTACAT/AATTATGT | 2 | 0.004 | 0.006 |
| AATTAGAT/AATTATCT | 1 | 0.002 | 0.003 |
| AATTGAGC/AATTGCTC | 1 | 0.002 | 0.003 |
| ACACACAG/CTGTGTGT | 1 | 0.002 | 0.003 |
| ACACACAT/ATGTGTGT | 7 | 0.014 | 0.020 |
| ACACACGC/CGTGTGTG | 1 | 0.002 | 0.003 |
| ACACACTC/AGTGTGTG | 1 | 0.002 | 0.003 |
| ACACAGAG/CTCTGTGT | 2 | 0.004 | 0.006 |
| ACACATAT/ATATGTGT | 3 | 0.006 | 0.009 |
| ACAGAGAG/CTCTCTGT | 10 | 0.020 | 0.029 |
| ACAGAGAT/ATCTCTGT | 1 | 0.002 | 0.003 |
| ACAGAGGC/CCTCTGTG | 1 | 0.002 | 0.003 |
| ACAGCTGC/AGCTGTGC | 1 | 0.002 | 0.003 |
| ACAGTGAG/ACTGTCTC | 1 | 0.002 | 0.003 |
| ACATACCT/AGGTATGT | 1 | 0.002 | 0.003 |
| ACATAGAT/ATCTATGT | 1 | 0.002 | 0.003 |
| ACATATAT/ATATATGT | 12 | 0.024 | 0.035 |
| ACATCCAT/ATGGATGT | 3 | 0.006 | 0.009 |
| ACATGCAT/ATGCATGT | 2 | 0.004 | 0.006 |
| ACCATCCC/ATGGTGGG | 1 | 0.002 | 0.003 |
| ACCATGAG/ATGGTCTC | 1 | 0.002 | 0.003 |
| ACCCGATG/ATCGGGTC | 1 | 0.002 | 0.003 |
| ACCCTATC/AGGGTGAT | 1 | 0.002 | 0.003 |
| ACGAGACT/AGTCTCGT | 1 | 0.002 | 0.003 |
| ACGCATGC/ATGCGTGC | 1 | 0.002 | 0.003 |
| ACGCGCGC/CGCGCGTG | 1 | 0.002 | 0.003 |
| ACGGATCC/ATCCGTGG | 1 | 0.002 | 0.003 |
| ACTCTCTC/AGAGAGTG | 2 | 0.004 | 0.006 |
| AGAGAGAT/ATCTCTCT | 1 | 0.002 | 0.003 |
| AGAGAGCG/CGCTCTCT | 2 | 0.004 | 0.006 |
| AGAGCATG/ATGCTCTC | 1 | 0.002 | 0.003 |
| AGAGGGGC/CCCCTCTG | 1 | 0.002 | 0.003 |
| AGAGGGGG/CCCCCTCT | 1 | 0.002 | 0.003 |
| AGATATAT/ATATATCT | 24 | 0.047 | 0.069 |
| AGATCATG/ATCTCATG | 1 | 0.002 | 0.003 |
| AGCATGGG/ATGCTCCC | 3 | 0.006 | 0.009 |
| AGGCGATC/ATCGCCTG | 1 | 0.002 | 0.003 |
| AAAAAAAAC/GTTTTTTTT | 7 | 0.014 | 0.020 |
| AAAAAAAAG/CTTTTTTTT | 16 | 0.031 | 0.046 |
| AAAAAAAAT/ATTTTTTTT | 13 | 0.026 | 0.037 |
| AAAAAAACC/GGTTTTTTT | 1 | 0.002 | 0.003 |
| AAAAAAATG/ATTTTTTTC | 2 | 0.004 | 0.006 |
| AAAAAAATT/AATTTTTTT | 3 | 0.006 | 0.009 |
| AAAAAACTG/AGTTTTTTC | 1 | 0.002 | 0.003 |
| AAAAAAGAG/CTCTTTTTT | 2 | 0.004 | 0.006 |
| AAAAAATTG/AATTTTTTC | 1 | 0.002 | 0.003 |
| AAAAACAAC/GTTGTTTTT | 2 | 0.004 | 0.006 |
| AAAAACTTC/AAGTTTTTG | 1 | 0.002 | 0.003 |
| AAAAAGAAG/CTTCTTTTT | 2 | 0.004 | 0.006 |
| AAAAATAAG/ATTTTTCTT | 2 | 0.004 | 0.006 |
| AAAAATAAT/ATTATTTTT | 7 | 0.014 | 0.020 |
| AAAAATAGC/ATTTTTGCT | 1 | 0.002 | 0.003 |
| AAAAATATG/ATATTTTTC | 1 | 0.002 | 0.003 |
| AAAAATGAT/ATCATTTTT | 1 | 0.002 | 0.003 |
| AAAAATTAC/AATTTTTGT | 1 | 0.002 | 0.003 |
| AAAAATTAG/AATTTTTCT | 2 | 0.004 | 0.006 |
| AAAAATTAT/AATTTTTAT | 1 | 0.002 | 0.003 |
| AAAAATTTT/AAAATTTTT | 2 | 0.004 | 0.006 |
| AAAACAAAG/CTTTGTTTT | 1 | 0.002 | 0.003 |
| AAAACAAAT/ATTTGTTTT | 1 | 0.002 | 0.003 |
| AAAACCCTC/AGGGTTTTG | 1 | 0.002 | 0.003 |
| AAAACCGAC/CGGTTTTGT | 1 | 0.002 | 0.003 |
| AAAAGAAGG/CCTTCTTTT | 2 | 0.004 | 0.006 |
| AAAAGCAAC/CTTTTGTTG | 2 | 0.004 | 0.006 |
| AAAAGGCAG/CCTTTTCTG | 2 | 0.004 | 0.006 |
| AAAAGTAGC/ACTTTTGCT | 1 | 0.002 | 0.003 |
| AAAATAAAC/ATTTTGTTT | 1 | 0.002 | 0.003 |
| AAAATAAAG/ATTTTCTTT | 1 | 0.002 | 0.003 |
| AAAATACAT/ATGTATTTT | 1 | 0.002 | 0.003 |
| AAAATATAC/ATATTTTGT | 2 | 0.004 | 0.006 |
| AAAATATCT/AGATATTTT | 2 | 0.004 | 0.006 |
| AAAATCATC/ATGATTTTG | 1 | 0.002 | 0.003 |
| AAAATTAAT/AATTTTATT | 1 | 0.002 | 0.003 |
| AAAATTACT/AATTTTAGT | 1 | 0.002 | 0.003 |
| AAACAAATT/AATTTGTTT | 1 | 0.002 | 0.003 |
| AAACATGTT/AACATGTTT | 1 | 0.002 | 0.003 |
| AAACATTGC/AATGTTTGC | 2 | 0.004 | 0.006 |
| AAACATTTC/AAATGTTTG | 2 | 0.004 | 0.006 |
| AAACTAGCT/AGCTAGTTT | 1 | 0.002 | 0.003 |
| AAACTTTCT/AAAGTTTAG | 1 | 0.002 | 0.003 |
| AAAGAAAGG/CCTTTCTTT | 3 | 0.006 | 0.009 |
| AAAGAAGAG/CTCTTCTTT | 1 | 0.002 | 0.003 |
| AAAGAAGAT/ATCTTCTTT | 2 | 0.004 | 0.006 |
| AAAGAAGGC/CCTTCTTTG | 1 | 0.002 | 0.003 |
| AAAGATGAG/ATCTTTCTC | 2 | 0.004 | 0.006 |
| AAAGCAATG/ATTGCTTTC | 1 | 0.002 | 0.003 |
| AAAGTTATG/AACTTTCAT | 1 | 0.002 | 0.003 |
| AAATAAATT/AATTTATTT | 2 | 0.004 | 0.006 |
| AAATAGAGG/ATTTCCTCT | 2 | 0.004 | 0.006 |
| AAATATATT/AATATATTT | 1 | 0.002 | 0.003 |
| AAATCAATC/ATTGATTTG | 1 | 0.002 | 0.003 |
| AAATCTTAT/AAGATTTAT | 1 | 0.002 | 0.003 |
| AAATTACAC/AATTTGTGT | 1 | 0.002 | 0.003 |
| AAATTCCTC/AATTTGAGG | 4 | 0.008 | 0.012 |
| AACAACAAT/ATTGTTGTT | 1 | 0.002 | 0.003 |
| AACAACAGC/CTGTTGTTG | 1 | 0.002 | 0.003 |
| AACAACATC/ATGTTGTTG | 1 | 0.002 | 0.003 |
| AACAACCAC/GGTTGTTGT | 1 | 0.002 | 0.003 |
| AACAAGAAG/CTTCTTGTT | 1 | 0.002 | 0.003 |
| AACAATAAT/ATTATTGTT | 1 | 0.002 | 0.003 |
| AACACACAC/GTGTGTGTT | 2 | 0.004 | 0.006 |
| AACACACCT/AGGTGTGTT | 1 | 0.002 | 0.003 |
| AACAGTTGC/AACTGTTGC | 1 | 0.002 | 0.003 |
| AACATGAAT/ATGTTATTC | 1 | 0.002 | 0.003 |
| AACCCGAAT/ATTCGGGTT | 2 | 0.004 | 0.006 |
| AACCTCCAC/AGGTTGTGG | 1 | 0.002 | 0.003 |
| AACCTCTTC/AAGAGGTTG | 1 | 0.002 | 0.003 |
| AACGACGAC/CGTCGTTGT | 1 | 0.002 | 0.003 |
| AACTTGAAG/AAGTTCTTC | 2 | 0.004 | 0.006 |
| AAGAAGATG/ATCTTCTTC | 2 | 0.004 | 0.006 |
| AAGAATCTT/AAGATTCTT | 1 | 0.002 | 0.003 |
| AAGAATGAG/ATTCTTCTC | 1 | 0.002 | 0.003 |
| AAGAGAAGT/ACTTCTCTT | 2 | 0.004 | 0.006 |
| AAGAGAATG/ATTCTCTTC | 2 | 0.004 | 0.006 |
| AAGAGGAGG/CCTCCTCTT | 1 | 0.002 | 0.003 |
| AAGATGATG/ATCATCTTC | 2 | 0.004 | 0.006 |
| AAGCAGAGG/CCTCTGCTT | 1 | 0.002 | 0.003 |
| AAGCATGTC/ACATGCTTG | 1 | 0.002 | 0.003 |
| AAGGAGAGG/CCTCTCCTT | 1 | 0.002 | 0.003 |
| AAGGTGAGG/ACCTTCCTC | 1 | 0.002 | 0.003 |
| AAGTACTAC/ACTTGTAGT | 1 | 0.002 | 0.003 |
| AAGTATGAG/ACTTCTCAT | 1 | 0.002 | 0.003 |
| AAGTCGTGT/ACACGACTT | 6 | 0.012 | 0.017 |
| AAGTGAGAG/ACTTCTCTC | 6 | 0.012 | 0.017 |
| AATAATAGT/ACTATTATT | 3 | 0.006 | 0.009 |
| AATAATATT/AATATTATT | 2 | 0.004 | 0.006 |
| AATAATTAT/AATTATTAT | 3 | 0.006 | 0.009 |
| AATAGAGAG/ATTCTCTCT | 1 | 0.002 | 0.003 |
| AATATATAG/ATATATTCT | 1 | 0.002 | 0.003 |
| AATATTATC/AATATTGAT | 2 | 0.004 | 0.006 |
| AATGTGATC/ACATTGATC | 1 | 0.002 | 0.003 |
| ACACACTGC/AGTGTGTGC | 5 | 0.010 | 0.014 |
| ACACGACCT/AGGTCGTGT | 1 | 0.002 | 0.003 |
| ACAGATGAT/ATCATCTGT | 2 | 0.004 | 0.006 |
| ACATGACCT/AGGTCATGT | 1 | 0.002 | 0.003 |
| ACATGAGGC/ATGTGCCTC | 1 | 0.002 | 0.003 |
| ACCAGGAGG/CCTCCTGGT | 1 | 0.002 | 0.003 |
| ACCATCTCT/AGAGATGGT | 1 | 0.002 | 0.003 |
| ACGAGCATG/ATGCTCGTC | 1 | 0.002 | 0.003 |
| AGCAGGATG/ATCCTGCTC | 1 | 0.002 | 0.003 |
| AGGATGATG/ATCATCCTC | 1 | 0.002 | 0.003 |
| AAAAAAAAAC/GTTTTTTTTT | 3 | 0.006 | 0.009 |
| AAAAAAAAAG/CTTTTTTTTT | 6 | 0.012 | 0.017 |
| AAAAAGAAAG/CTTTCTTTTT | 1 | 0.002 | 0.003 |
| AAAAAGAAGG/CCTTCTTTTT | 1 | 0.002 | 0.003 |
| AAAAATAAAT/ATTTATTTTT | 4 | 0.008 | 0.012 |
| AAAACGAAAG/CGTTTTCTTT | 1 | 0.002 | 0.003 |
| AAAACTCAAG/AGTTTTCTTG | 1 | 0.002 | 0.003 |
| AAAACTTTAT/AAAGTTTTAT | 1 | 0.002 | 0.003 |
| AAAAGGATTT/AAATCCTTTT | 2 | 0.004 | 0.006 |
| AAAATTATCC/AATTTTGGAT | 1 | 0.002 | 0.003 |
| AAACCAAACT/AGTTTGGTTT | 1 | 0.002 | 0.003 |
| AAAGAAGAAG/CTTCTTCTTT | 1 | 0.002 | 0.003 |
| AAAGAGAATG/ATTCTCTTTC | 1 | 0.002 | 0.003 |
| AAAGAGACAG/CTCTTTCTGT | 1 | 0.002 | 0.003 |
| AAAGAGAGAG/CTCTCTCTTT | 2 | 0.004 | 0.006 |
| AAAGGATGGG/ATCCTTTCCC | 1 | 0.002 | 0.003 |
| AAAGTTAGGC/AACTTTGCCT | 7 | 0.014 | 0.020 |
| AAATAATGTT/AACATTATTT | 1 | 0.002 | 0.003 |
| AAATACATAT/ATATGTATTT | 1 | 0.002 | 0.003 |
| AAATATGTCC/ACATATTTGG | 1 | 0.002 | 0.003 |
| AAATCTCCAT/AGATTTATGG | 1 | 0.002 | 0.003 |
| AAATCTTATT/AAGATTTAAT | 1 | 0.002 | 0.003 |
| AAATTAACCT/AATTTAGGTT | 1 | 0.002 | 0.003 |
| AAATTATATT/AATATAATTT | 2 | 0.004 | 0.006 |
| AACAATTATT/AATAATTGTT | 8 | 0.016 | 0.023 |
| AACAATTCAT/AATTGTTATG | 2 | 0.004 | 0.006 |
| AACATAATAC/ATGTTGTATT | 1 | 0.002 | 0.003 |
| AACATAGTCT/ACTATGTTAG | 1 | 0.002 | 0.003 |
| AACGCAGCAT/ATGCTGCGTT | 3 | 0.006 | 0.009 |
| AACTGACCCG/AGTTCGGGTC | 4 | 0.008 | 0.012 |
| AAGATAAGCC/ATCTTGGCTT | 1 | 0.002 | 0.003 |
| AAGATAGGTC/ACCTATCTTG | 2 | 0.004 | 0.006 |
| AATAATAATT/AATTATTATT | 1 | 0.002 | 0.003 |
| AATATATAGT/ACTATATATT | 1 | 0.002 | 0.003 |
| AATCATGATT/AATCATGATT | 1 | 0.002 | 0.003 |
| AATTACTCAT/AATTATGAGT | 1 | 0.002 | 0.003 |
| AATTAGATAT/AATTATATCT | 2 | 0.004 | 0.006 |
| AATTCGGGTC/AATTGACCCG | 3 | 0.006 | 0.009 |
| ACACTCTCTC/AGAGAGTGTG | 2 | 0.004 | 0.006 |
| ACATACATAT/ATATGTATGT | 2 | 0.004 | 0.006 |
| ACATATATAT/ATATATATGT | 1 | 0.002 | 0.003 |
| ACATGATGAG/ATCATGTCTC | 1 | 0.002 | 0.003 |
| ACCCGATCCG/ATCGGGTCGG | 1 | 0.002 | 0.003 |
| ACCTGAGAGC/AGGTGCTCTC | 1 | 0.002 | 0.003 |
| AGAGAGAGGG/CCCTCTCTCT | 1 | 0.002 | 0.003 |
| AGATAGATAT/ATATCTATCT | 2 | 0.004 | 0.006 |

**Table S4 .** *In silico* PCR result with *C. clementina.*

|  | Mono morphic | Poly morphic | Total VPCR  positive SSR | Total Primer  Tested | % VPCR  validation |
| --- | --- | --- | --- | --- | --- |
| Di | 1781 | 1413 | 3194 | 10567 | 30.23 |
| Tri | 1208 | 631 | 1839 | 6051 | 30.39 |
| Tetra | 412 | 237 | 649 | 1858 | 34.93 |
| Penta | 382 | 250 | 632 | 2000 | 31.60 |
| Hexa | 158 | 116 | 274 | 772 | 35.49 |
| Total (%) | 3941(59.82) | 2647(40.18) | 6588 | 21248 | 31.01 |

**Table S5 .** Results of Microsatellite Search.

| Plant Species | | Total size of examined sequences (bp): | Total number of identified SSRs: | One SSR found in: (Mbp genome) | SSRs Frequency (SSR/Mbp): | Genome size (Mbp) |
| --- | --- | --- | --- | --- | --- | --- |
|  | *A..thaliana* | 119667750 | 12787 | 0.009358548 | 106.8541859 | 119.66775 |
|  | *C. papaya* | 342680090 | 63583 | 0.005389492 | 185.5462335 | 342.68009 |
|  | *C. sativus* | 203058019 | 32273 | 0.006291885 | 158.9348707 | 203.058019 |
| Dicot | *M. domestica* | 881278625 | 126263 | 0.006979706 | 143.2725093 | 881.278625 |
|  | *M. truncatula* | 307481907 | 39581 | 0.007768422 | 128.7262733 | 307.481907 |
|  | *P. trichocarpa* | 417137944 | 73496 | 0.005675655 | 176.1911163 | 417.137944 |
|  | *C. sinensis* | 347267366 | 50846 | 0.00682979 | 146.417444 | 347.267366 |
|  | *C. clementina* | 295550349 | 48284 | 0.006121083 | 163.3697952 | 295.550349 |
|  | *S. bicolor* | 738540932 | 47570 | 0.015525 | 64.41078 | 738.5409 |
| Monocot | *Z. manize* | 2066432718 | 62915 | 0.032844834 | 30.44618847 | 2066.432718 |
|  | *O. sativa* | 374471240 | 41342 | 0.00905789 | 110.401002 | 374.47124 |

**Table S6 .** Distribution to different repeat type classes among the 11 plant species.

|  | Mono- | Di- | Tri- | Tetra- | Penta- | Hexa- | Hepta- | Octa- | Nova- | Deca- |
| --- | --- | --- | --- | --- | --- | --- | --- | --- | --- | --- |
| *A. thaliana* | 4303 | 4602 | 2350 | 169 | 351 | 178 | 691 | 82 | 39 | 22 |
| *C. papaya* | 10937 | 38016 | 6511 | 2099 | 2978 | 1201 | 1391 | 312 | 80 | 58 |
| *C. sativus* | 3505 | 15330 | 5573 | 1521 | 1758 | 1148 | 2337 | 764 | 265 | 72 |
| *M. domestica* | 25704 | 66577 | 14552 | 3718 | 4399 | 1696 | 6134 | 2209 | 888 | 386 |
| *M. truncatula* | 17602 | 13257 | 4396 | 1079 | 1455 | 664 | 882 | 178 | 50 | 18 |
| *P. trichocarpa* | 12697 | 33719 | 14088 | 3178 | 3977 | 2355 | 2864 | 433 | 129 | 56 |
| *C. sinensis* | 13828 | 16713 | 10162 | 2826 | 2960 | 1194 | 2183 | 704 | 193 | 83 |
| *C. clementina* | 13373 | 16207 | 9469 | 2679 | 2834 | 1074 | 1873 | 551 | 161 | 63 |
| *S. bicolor* | 3790 | 18937 | 11895 | 5368 | 3162 | 2648 | 1397 | 170 | 134 | 69 |
| *Z. manize* | 7986 | 25844 | 15080 | 3316 | 4861 | 2682 | 2153 | 429 | 424 | 140 |
| *O. sativa* | 3152 | 17829 | 11948 | 2579 | 2963 | 1398 | 968 | 321 | 91 | 93 |

**Table S7 .** Chi square and correlation analysis.

| SSR repeat class I and class II | | | | |
| --- | --- | --- | --- | --- |
|  | Chi square value | 611.489124 | *** |  |
|  | P-value | 5.3072E-135 |  |  |
|  | α=0.05, df =1 |  |  |  |
|  | Correlation r = | -0.86835 |  |  |
| SSR distribution among the chromosome | | | | |
|  | Chi square value = | 6094.8869 | *** |  |
|  | P-value = | 0.0000 |  |  |
|  | α=0.05, df =9 |  |  |  |
|  | Correlation r = | -0.7441 | (Chromosome size Vs SSR frequency) | |
| Correlation Genome size Vs SSR frequency of 11 plat species | | | | |
|  | Correlation r = | -0.70649493 |  |  |
| Correlation Repeat Class length Vs Repeat Count | | | | |
|  | Athaliana | -0.814550027 |  |  |
|  | Cpapaya | -0.627899363 |  |  |
|  | Csativus | -0.638214207 |  |  |
|  | Mdomestica | -0.666013383 |  |  |
|  | Mtruncatula | -0.800644858 |  |  |
|  | Ptrichocarpa | -0.732002683 |  |  |
|  | Csinensis | -0.868349198 |  |  |
|  | Cclementina | -0.86674184 |  | |
|  | Sbicolor | -0.709220386 |  | |
|  | Zmanize | -0.729831307 |  | |
|  | Osativa | -0.667397659 |  | |

**Table S8 .** Strand specificity of perfect microsatellites in *C. sinensis* transcribed regions.

| Repeat motif | Total  Number | Type | Number | Type | Number | Chi  square | P-Value |
| --- | --- | --- | --- | --- | --- | --- | --- |
| **5'UTR** |  |  |  |  |  |  |  |
| A/T | 424 | A | 286 | T | 138 | 51.66 | 0.0000 |
| AG/CT | 481 | AG | 107 | CT | 153 | 105.94 | 0.0000 |
| AT/AT | 84 | AT | 40 | AT | 44 | 0.19 | 0.6625 |
| AC/GT | 79 | AC | 28 | GT | 51 | 6.70 | 0.0097 |
| AAG/CTT | 74 | AAG | 47 | CTT | 27 | 5.41 | 0.0201 |
| AAT/ATT | 83 | AAT | 40 | ATT | 43 | 0.11 | 0.7419 |
| AAAG/CTTT | 33 | AAAG | 18 | CTTT | 15 | 0.27 | 0.6015 |
| AAAT/ATTT | 30 | AAAT | 16 | ATTT | 14 | 0.13 | 0.7150 |
| AAAAG/CTTTT | 21 | AAAAG | 17 | CTTTT | 4 | 8.05 | 0.0046 |
|  |  |  |  |  |  |  |  |
| **3'UTR** |  |  |  |  |  |  |  |
| A/T | 514 | A | 170 | T | 344 | 58.90 | 0.0000 |
| AT/AT | 149 | AT | 83 | AT | 66 | 1.94 | 0.1637 |
| AG/CT | 98 | AG | 43 | CT | 55 | 1.47 | 0.2254 |
| AC/GT | 75 | AC | 30 | GT | 45 | 3.00 | 0.0833 |
| AAT/ATT | 80 | AAT | 30 | ATT | 50 | 5.00 | 0.0253 |
| AAG/CTT | 30 | AAG | 11 | CTT | 19 | 2.13 | 0.1441 |
| AAAT/ATTT | 26 | AAAT | 8 | ATTT | 18 | 3.85 | 0.0499 |
|  |  |  |  |  |  |  |  |
| **CDS** |  |  |  |  |  |  |  |
| A/T | 20 | A | 9 | T | 11 | 0.20 | 0.6547 |
| AG/CT | 45 | AG | 13 | CT | 32 | 8.02 | 0.0046 |
| AAG/CTT | 308 | AAG | 219 | CTT | 89 | 54.87 | 0.0000 |
| AGC/CTG | 254 | AGC | 200 | CTG | 54 | 83.92 | 0.0000 |
| ATC/ATG | 183 | ATC | 68 | ATG | 115 | 12.07 | 0.0005 |
| AGG/CCT | 159 | AGG | 101 | CCT | 58 | 11.63 | 0.0006 |
| ACC/GGT | 122 | ACC | 78 | GGT | 44 | 9.48 | 0.0021 |
| AAC/GTT | 116 | AAC | 90 | GTT | 26 | 35.31 | 0.0000 |
| AAT/ATT | 95 | AAT | 78 | ATT | 17 | 39.17 | 0.0000 |
| ACG/CGT | 42 | ACG | 35 | CGT | 7 | 18.67 | 0.0000 |
| CCG/CGG | 37 | CCG | 17 | CGG | 20 | 0.24 | 0.6219 |

**Table S9 .** SSR distribution in different genomic fraction on Sweet orange genome.

| Unit size |  |  | Number of SSRs | |  |  |
| --- | --- | --- | --- | --- | --- | --- |
|  | Introns | 3'-UTR | 5'-UTR | CDS | Exon | Intergenic |
| 1 | 5038 | 530 | 435 | 25 | 2124 | 12180 |
| 2 | 4809 | 322 | 644 | 65 | 2279 | 14784 |
| 3 | 1892 | 150 | 266 | 1325 | 2267 | 8385 |
| 4 | 930 | 50 | 99 | 2 | 349 | 2578 |
| 5 | 591 | 63 | 100 | 9 | 412 | 2612 |
| 6 | 242 | 22 | 53 | 228 | 437 | 903 |
| 7 | 534 | 49 | 76 | 7 | 261 | 1936 |
| 8 | 149 | 15 | 32 | 7 | 83 | 614 |
| 9 | 30 | 5 | 3 | 12 | 27 | 161 |
| 10 | 7 | 1 | 5 | 0 | 13 | 67 |
| Total | 14222 | 1207 | 1713 | 1680 | 8252 | 44220 |
|  |  |  |  |  |  |  |
| % of the total SSR occurred | 19.9484 | 1.6930 | 2.4027 | 2.3564 | 11.5746 | 62.0249 |
| Frequency (SSR/Mbp) | 185.7580 | 177.6155 | 472.0979 | 30.2455 | 102.2037 | 156.8700 |

**Table S10 .** Summary of the wet lab experiment.

|  | No of  Individual  Citrus sp. | No of  tested  marker | Amplified  marker | % of  transferability |
| --- | --- | --- | --- | --- |
| Sweet orange | 4 | 950 | 578 | 60.84 |
| C grandis | 3 | 950 | 533 | 56.11 |
| Lemon | 2 | 950 | 547 | 57.58 |
| Kumquat | 5 | 950 | 501 | 52.74 |
| Trifoliata | 2 | 950 | 479 | 50.42 |
| Total | 16 | 950 |  |  |
| Over all amplified (%) | 609(64.11) | |  |  |
| Polymorphic (%) | 534(56.21) | |  |  |
| Over all transferable (%) | 536(56.42) | |  |  |
|  | Tested | No of  Amplified  Marker (%) | No of  Transferable  Marker (%) | Polymorphic  Marker (%) |
| Coding SSR | 520 | 348(66.92) | 327 (62.88) | 302(58.07) |
| Non Coding SSR | 430 | 261(60.70) | 209 (48.60) | 232(53.95) |
